# Highly Efficient Proton Conduction in the Metal–Organic Framework Material MFM-300(Cr)·SO_4_(H_3_O)_2_

**DOI:** 10.1021/jacs.2c04900

**Published:** 2022-07-01

**Authors:** Jin Chen, Qingqing Mei, Yinlin Chen, Christopher Marsh, Bing An, Xue Han, Ian P. Silverwood, Ming Li, Yongqiang Cheng, Meng He, Xi Chen, Weiyao Li, Meredydd Kippax-Jones, Danielle Crawshaw, Mark D. Frogley, Sarah J. Day, Victoria García-Sakai, Pascal Manuel, Anibal J. Ramirez-Cuesta, Sihai Yang, Martin Schröder

**Affiliations:** † Department of Chemistry, The University of Manchester, Manchester M13 9PL, United Kingdom; ‡ ISIS Neutron and Muon Source, Rutherford Appleton Laboratory, Didcot OX11 0QX, United Kingdom; § Faculty of Engineering, University of Nottingham, Nottingham NG7 2RD, United Kingdom; ∥ Neutron Scattering Division, Neutron Sciences Directorate, Oak Ridge National Laboratory, Oak Ridge, Tennessee 37831, United States; ⊥ Diamond Light Source, Harwell Science Campus, Oxfordshire OX11 0DE, United Kingdom

## Abstract

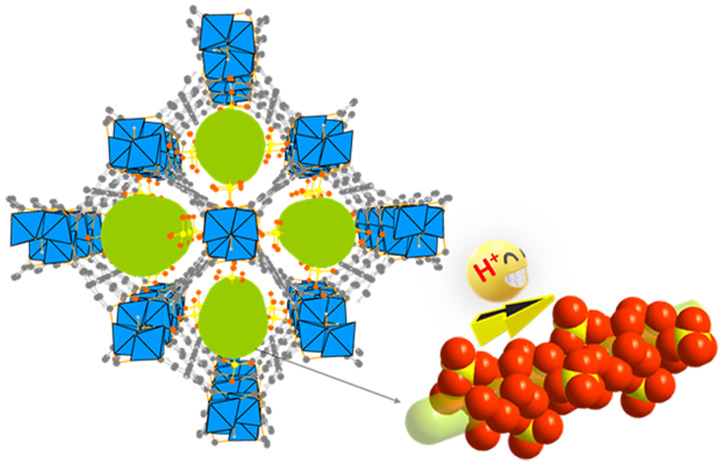

The development of materials showing rapid proton conduction with a low activation energy and stable performance over a wide temperature range is an important and challenging line of research. Here, we report confinement of sulfuric acid within porous MFM-300(Cr) to give MFM-300(Cr)·SO_4_(H_3_O)_2_, which exhibits a record-low activation energy of 0.04 eV, resulting in stable proton conductivity between 25 and 80 °C of >10^–2^ S cm^–1^. *In situ* synchrotron X-ray powder diffraction (SXPD), neutron powder diffraction (NPD), quasielastic neutron scattering (QENS), and molecular dynamics (MD) simulation reveal the pathways of proton transport and the molecular mechanism of proton diffusion within the pores. Confined sulfuric acid species together with adsorbed water molecules play a critical role in promoting the proton transfer through this robust network to afford a material in which proton conductivity is almost temperature-independent.

Proton exchange membrane (PEM) fuel cells enable the utilization of hydrogen for portable applications.^1^ The development of PEM materials showing high and stable proton conductivity over a wide temperature range is of critical importance to the operation of fuel cells,^2^ and a wide variety of proton conductors, such as Nafion, metal oxides, and mesoporous silica, have been investigated.^3^ Most of these materials show an activation energy above 0.1 eV and thus have drawbacks such as prolonged start-up time for automobile applications owing to restricted molecular dynamics.^4^

Over the past decade, metal–organic framework (MOF) materials have emerged as promising targets for PEM applications due to their designable functionality and proton conductivity that can be comparable to Nafion, a benchmark material in this area.^5^ To further improve the proton conductivity of MOFs, two strategies have been employed: (i) increasing the density of active protons within the framework by introducing functional or acidic groups^6^ and (ii) increasing the mobility of the active protons in the pore by constructing hydrogen-bonded networks as efficient pathways for proton transport.^7^ Among these, introducing sulfuric acid into MOFs is a particularly promising approach to enhancing proton conductivity because of its strong acidity and multiple hydrogen donor/acceptor sites that promote the formation of hydrogen-bonded networks.^8^ However, to date, all MOF-based proton conductors exhibit an activation energy above 0.1 eV, rendering their proton conductivity highly temperature-dependent.^9^

The crystalline nature of MOFs enables interrogation of the mechanisms of proton conduction, thus providing key insights into the design of new PEM materials with improved performance.^9^ Indeed, this is an overwhelming strength of MOFs compared with amorphous polymer-based materials such as Nafion.^5^ X-ray crystallography and pulsed field gradient NMR spectroscopy have been used to investigate the hydrogen-bonded network and the details of proton diffusion within MOFs.^10^ While the former is subject to inherent uncertainties in terms of the location of protons, the latter can underestimate the diffusion rate of protons.^11^ Quasielastic neutron scattering (QENS) is a powerful technique to study the molecular dynamics spanning a broad time (10^–13^–10^–7^ s) and length scale (10^–10^–10^–7^ m)^12^ and is especially useful for the investigation of the dynamics of protons due to the large incoherent cross-section of hydrogen for neutron scattering.^13^ However, this technique has only been used to probe the diffusion of protons in MOFs in very limited cases.^6b,11,14^

Herein, we report the postsynthetic modification of a highly robust MFM-300(Cr)·*x*H_2_O by reaction with chlorosulfonic acid. The resultant MFM-300(Cr)·SO_4_(H_3_O)_2_ exhibits a record-low activation energy for an MOF of 0.04 eV and a high proton conductivity of >10^–2^ S cm^–1^ over a wide temperature range 25–80 °C. High-resolution synchrotron X-ray powder diffraction (SXPD) and neutron powder diffraction (NPD) confirm the formation of a helical hydrogen-bonded network composed of confined SO_4_^2–^, H_3_O^+^, and H_2_O species within the pores of MFM-300(Cr)·SO_4_(H_3_O)_2_. An analysis of the proton dynamics within MFM-300(Cr)·SO_4_(H_3_O)_2_ by QENS and molecular dynamics (MD) simulation has revealed that the protons can access every point of the hydrogen-bonded network in the channel via a Hall–Ross jump diffusion mechanism and shows temperature-independent diffusion, which validates the ultralow activation energy of MFM-300(Cr)·SO_4_(H_3_O)_2_.

MFM-300(Cr), [Cr_2_(OH)_2_L]·5H_2_O (H_4_L = biphenyl-3,3′,5,5′-tetracarboxylic acid),^15^ was selected for the postsynthetic modification with chlorosulfonic acid owing to its ultrahigh stability (Figure S1). MFM-300(Cr) was reacted with chlorosulfonic acid in CH_2_Cl_2_ for 2 h and then washed with fresh CH_2_Cl_2_ to yield the modified material, MFM-300(Cr)·SO_4_(H_3_O)_2_. Powder X-ray diffraction confirms no structural change upon the postsynthetic modification (Figure S2), and the molar ratio between chromium and sulfur is determined to be 1.0:0.36 by elemental analysis, consistent with that (1.0:0.34) determined by thermal gravimetric analysis (TGA). The images from scanning electron microscopy (SEM) confirm the retention of a rodlike morphology for the material upon postsynthetic modification, and EDX analysis indicates a homogeneous distribution of the S-containing species throughout the material (Figure S3). The FTIR spectrum of MFM-300(Cr)·SO_4_(H_3_O)_2_ shows new peaks at 1002, 1110, and 1226 cm^–1^ compared to MFM-300(Cr) assigned to ν(S—O) stretching, and symmetric and asymmetric ν(S=O) stretching vibrations, respectively (Figure S4).^16^ TGA profiles show additional weight loss at 450–500 °C in MFM-300(Cr)·SO_4_(H_3_O)_2_, and an FTIR analysis of the species emitted at ∼470 °C confirms the presence of SO_2_ (Figures S5 and S6); no SO_2_ was observed on heating the parent MFM-300(Cr). A ^1^H NMR spectroscopic analysis of the digested sample of MFM-300(Cr)·SO_4_(H_3_O)_2_ confirmed the full retention of the chemical integrity of the organic linker (Figure S7). Water vapor sorption isotherms (Figure S17) and *in situ* FTIR spectroscopy as a function of H_2_O adsorption suggest that SO_4_^2–^ species in the channels interact with the bridging hydroxyl groups in MFM-300(Cr)·SO_4_(H_3_O)_2_ (Figure S20).

Rietveld refinement of the SXPD data for MFM-300(Cr)·SO_4_(H_3_O)_2_ confirms full retention of the framework structure of MFM-300(Cr) (Figure 1a, Figure S8 and Table S1). Sulfuric acid is located within the channels, forms hydrogen bonds to the bridging −OH groups [O···O = 2.63(9) Å; Figure 1b,c], and is further bridged by water molecules to form an extensive hydrogen-bonded network [O···O = 2.3–5.7 Å] (Figure 1d). This structural model is entirely consistent with that obtained from refinements of NPD data and by *in situ* FTIR analysis (Figures S19 and S20). Such a network provides multiple pathways for proton transport, which is critical to drive proton conduction within solid-state materials.^17^

**Figure 1:**
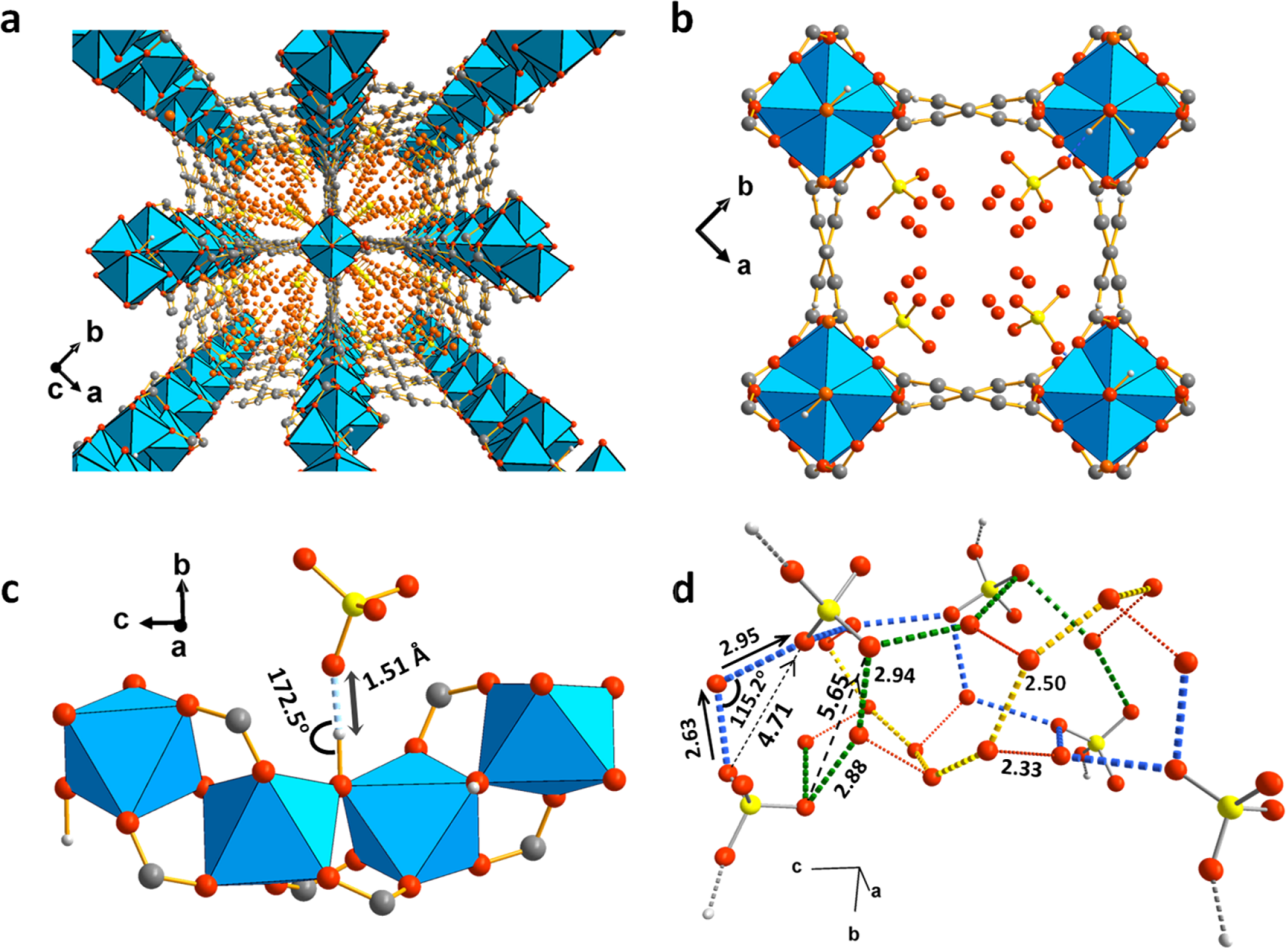
Structure of MFM-300(Cr)·SO_4_(H_3_O)_2_. (a) View along the crystallographic *c*-axis. (b) View of packing of guest molecules along the channel along the *c*-axis. (c) Enlarged view of the interaction between SO_4_^2–^ and the −OH group. (d) View of the hydrogen-bonded network in the channel. Dashed lines illustrate potential paths for proton transport. Distances are in Å. Chromium, blue; carbon, dark gray; oxygen, red; sulfur, yellow; hydrogen, light gray (partially omitted).

The proton conductivity of MFM-300(Cr) and MFM-300(Cr)·SO_4_(H_3_O)_2_ was analyzed by AC impedance spectroscopy (Figure 2a and Figure S9). Both materials show water-dependent proton conductivity (Figure S12). The proton conductivity of MFM-300(Cr)·SO_4_(H_3_O)_2_ was measured to be 1.26 × 10^–2^ S cm^–1^ at 25 °C and 99% relative humidity (RH), which is 3 orders of magnitude higher than that of the parent MOF (5.72 × 10^–6^ S cm^–1^ at 25 °C and 99% RH). This can be attributed to the confined sulfuric acid that provides additional active protons and create multiple hydrogen bonds in cooperation with the confined water molecules in the channel to promote proton transport. The proton conductivity of MFM-300(Cr)·SO_4_(H_3_O)_2_ at 25 °C is within the range of superprotonic conductivity^18^ and is comparable to that of the best-performing MOFs reported to date.^8,18−20^ Interestingly, the proton conductivity of MFM-300(Cr)·SO_4_(H_3_O)_2_ was found to be almost independent of temperature with an ultralow activation energy of 0.04 eV, representing the lowest value for MOF-based proton conductors reported to date (Figure 2b and Table S2).^9^ A low activation energy translates to stable proton conductivity over a wide range of temperature.^4a,5^ In contrast, MFM-300(Cr) shows an activation energy of 0.51 eV, consistent with a vehicular mechanism, where proton transport is enabled solely by the adsorbed water molecules within the channels.^21^ The activation energy was also determined at various RH conditions for MFM-300(Cr)·SO_4_(H_3_O)_2_, and this suggested that the pathway for proton hopping can be maintained even in a medium RH environment (Figure S16). Proton conductivity of MFM-300(Cr)·SO_4_(H_3_O)_2_ was monitored over three cycles of heating and cooling from 25 to 80 °C under 99% RH, and no loss of proton conductivity was observed (Figure 2c), with the sample retaining its structure throughout (Figure S18a). Importantly, the structure and proton conductivity of MFM-300(Cr)·SO_4_(H_3_O)_2_ are retained after two years of being stored under ambient conditions (Figure 2d and Figure S18b), thus demonstrating its high stability.

**Figure 2:**
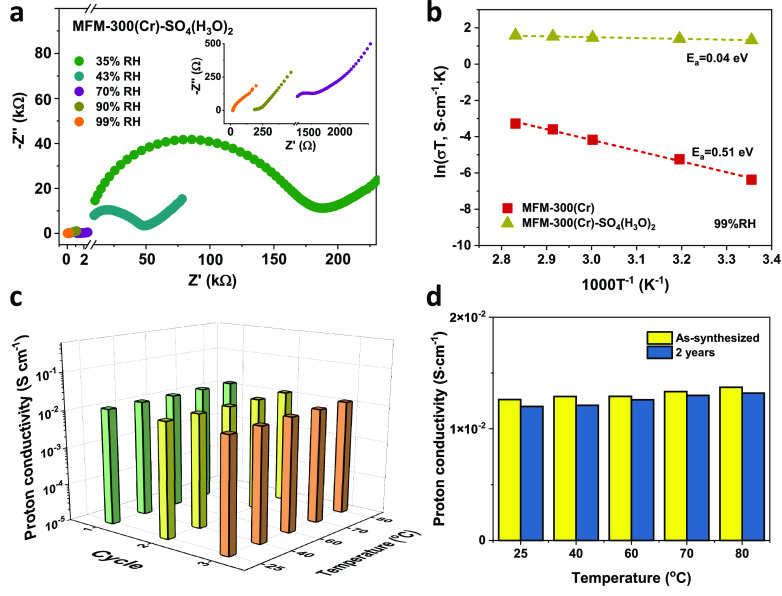
(a) Nyquist plots for MFM-300(Cr)·SO_4_(H_3_O)_2_ at room temperature (the inset is the enlarged view of results at high RH). (b) Arrhenius plots of proton conductivity for MFM-300(Cr) and MFM-300(Cr)·SO_4_(H_3_O)_2_ under 99% RH. (c) Comparison of proton conductivity of MFM-300(Cr)·SO_4_(H_3_O)_2_ over three cycles of heating–cooling processes under 99% RH. (d) Proton conductivity for as-synthesized MFM-300(Cr)·SO_4_(H_3_O)_2_ and MFM-300(Cr)·SO_4_(H_3_O)_2_ that has been stored in an ambient environment for two years.

To investigate the conduction mechanism, QENS spectra were measured for both MFM-300(Cr) and MFM-300(Cr)·SO_4_(H_3_O)_2_ under 99% RH and at temperatures between 0 and 80 °C (Figure 3a). The half-width at half-maximum (HWHM) profiles of MFM-300(Cr)·SO_4_(H_3_O)_2_ can be best fitted to a Hall–Ross model (eq 1)^13b^ (Equations 1 and S1–S4, and Figures 3b and S13):
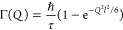
1where Γ is the HWHM of QENS peak, *Q* is the scattering vector, and *l* and τ are the mean jump length and relaxation time of the diffusing particles, respectively. The diffusion coefficient *D* can be derived from eq 2:
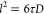
2

**Figure 3:**
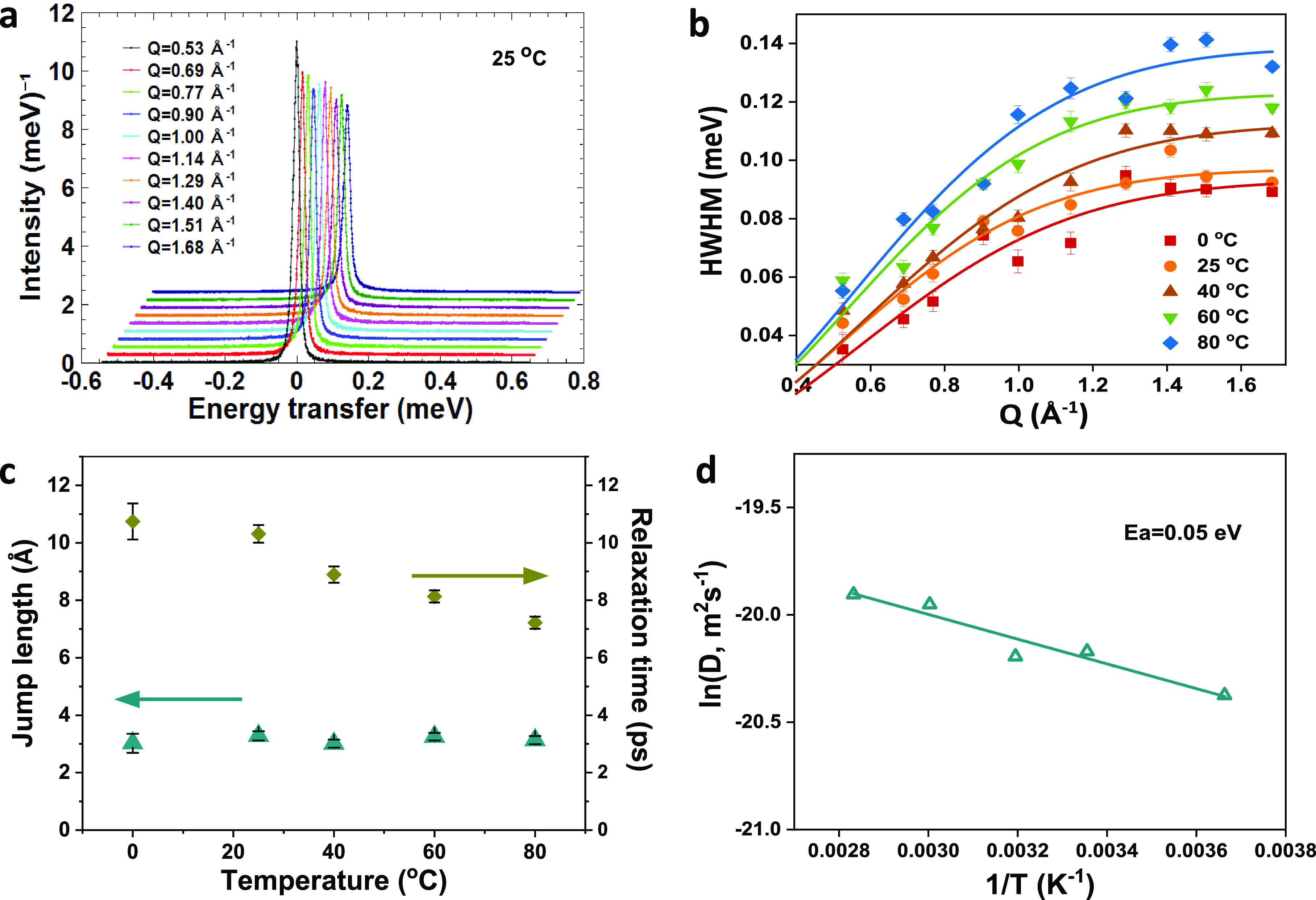
(a) QENS spectra for MFM-300(Cr)·SO_4_(H_3_O)_2_ measured at 25 °C. (b) HWHM of QENS spectra as a function of *Q*, fitted with the Hall–Ross model for MFM-300(Cr)·SO_4_(H_3_O)_2_ at different temperatures. (c) Jump length and relaxation time as a function of temperature for MFM-300(Cr)·SO_4_(H_3_O)_2_. (d) Arrhenius plot of the diffusion coefficient derived from QENS analysis for MFM-300(Cr)·SO_4_(H_3_O)_2_.

This suggests that protons in the sample jump freely between sites of the hydrogen-bonded networks.^12,22^ The mean jump length, *l*, is determined to be 3.0–3.1 Å at 0–80 °C (Figure 3c), consistent with the intermolecular distances between sulfuric acid and water molecules in the channel (Figure 1d). In addition, *l* is found to be temperature-independent, and the observed relaxation times and diffusion coefficients experience only small changes across the temperature range. The activation energy obtained from the QENS analysis is 0.05 eV, in excellent agreement with that derived from impedance analysis (Figure 3d). Interestingly, a theoretical study has suggested that the activation energy of proton transfer between sulfuric acid species can be lowered significantly by water that allows protons to hop through the hydrogen-bonded network in a “rocking” mode, yielding a predicted activation energy as low as 0.06 eV.^23^ The self-diffusion coefficient of the protons in MFM-300(Cr)·SO_4_(H_3_O)_2_ was calculated to be 1.8 × 10^–9^ and 2.3 × 10^–9^ m^2^ s^–1^ at 25 and 80 °C, respectively (Figure S15), similar to those reported for MOFs with high proton conductivities^9^ such as UiO-66(Zr)-(CO_2_H)_2_ [*D*(*H*) = 1.1 × 10^–9^ m^2^ s^–1^ at 25 °C]^14a^ and defective UiO-66 [*D*(*H*) = 4.0 × 10^–11^ m^2^ s^–1^ at 25 °C],^10b^ determined by QENS and NMR spectroscopy, respectively. MFM-300(Cr) shows a lower *D* value of 2.2 × 10^–10^ m^2^ s^–1^ at 25 °C, consistent with its low proton conductivity. The process of proton transportation is also demonstrated and visualized by MD simulation (Figure S21). Specifically, protons in the SO_4_^2–^-H_3_O^+^-H_2_O hydrogen-bonded network show high mobility, and transportation is achieved by proton jumping between neighboring sites and reorientation (by rocking motions) of the H_2_O/H_3_O/SO_4_ species.

The broadening of the QENS peak at 0 °C for MFM-300(Cr) within a given range of *Q* is nearly constant (1–2 μeV; Figure S14), lower than the resolution of the instrument (∼17 μeV),^14b^ suggesting that the diffusion of protons in MFM-300(Cr) is too slow to be detected at 0 °C. This is because the movement of water molecules, which serve as vehicles to assist the proton transfer in MFM-300(Cr), is significantly hindered at 0 °C. However, MFM-300(Cr)·SO_4_(H_3_O)_2_ exhibits a remarkable diffusion coefficient of 1.4 × 10^–9^ m^2^ s^–1^ at 0 °C, attributed to the extensive hydrogen-bonded network composed of both sulfuric species and water molecules, allowing protons to transfer more efficiently with an ultralow energy barrier.

## Supporting Information Available

Synthesis procedure, characterization, impedance spectra, QENS analysis, SXPD and NPD refinements, *in situ* FTIR, and MD simulation (PDF)
