# Supplementary material for: Highly Efficient Proton Conduction in the Metal–Organic Framework Material MFM-300(Cr)·SO4(H3O)2
Source: J Am Chem Soc. 2022 Jul 1;144(27):11969–74. doi: 10.1021/jacs.2c04900 (PMC9348827; doi:10.1021/jacs.2c04900)
Supplement: Supplementary file 1 — ja2c04900_si_001.pdf [file ja2c04900_si_001.pdf]

Supplementary Information

Highly efficient proton conduction in the metal-organic framework material MFM-300(Cr).$SO_4(H_3O)_2$

Jin Chen,[1] Qingqing Mei,[1] Yinlin Chen,[1] Christopher Marsh,[1] Bing An,[1] Xue Han,[1] Ian P. Silverwood,[2] Ming Li,[3] Yongqiang Cheng,[4] Meng He,[1] Xi Chen,[1] Weiyao Li,[1] Meredydd Kippax-Jones,[1,5] Danielle Crawshaw,[1] Mark D. Frogley,[5] Sarah J. Day,[5] Victoria García-Sakai,[2] Pascal Manuel,[2] Anibal J. Ramirez-Cuesta[4], Sihai Yang*[1] and Martin Schröder*[1]

[1]Department of Chemistry, The University of Manchester, Manchester, M13 9PL, UK

[2]ISIS Neutron and Muon Source, Rutherford Appleton Laboratory, Didcot, OX11 0QX, UK

[3]Faculty of Engineering, University of Nottingham, Nottingham NG7 2RD, UK

[4]Neutron Scattering Division, Neutron Sciences Directorate, Oak Ridge National Laboratory, Oak Ridge, TN 37831, USA

[5]Diamond Light Source, Harwell Science Campus, Oxfordshire OX11 0DE, UK

Content

1. Materials and Methods S4

1.1 Synthesis S4

1.2 General characterization S4

1.3 Structure determination and refinements of synchrotron XPD data S5

1.4 Impedance measurements S5

1.5 Quasi-elastic neutron scattering (QENS) S5

1.6 Neutron powder diffraction (NPD) experiment and refinement S6

1.7 *In situ* infrared spectroscopy S7

1.8 Molecular dynamics (MD) simulation S7

2. Supplementary Figures and Tables S8

Figure S1. Structure of MFM-300(Cr) S8

Figure S2. PXRD patterns of MFM-300(Cr) and MFM-300(Cr)-$SO_4(H_3O)_2$ S8

Figure S3. SEM images and elemental mapping of MFM-300(Cr)-$SO_4(H_3O)_2$ S9

Figure S4. Infrared spectra of MFM-300(Cr) and MFM-300(Cr)-$SO_4(H_3O)_2$ S10

Figure S5. TGA profiles of MFM-300(Cr) and MFM-300(Cr)-$SO_4(H_3O)_2$ S11

Figure S6. TGA-MS profiles of MFM-300(Cr) and MFM-300(Cr)-$SO_4(H_3O)_2$ S12

Figure S7. ^{1}H NMR spectra of digested MFM-300(Cr) and MFM-300(Cr)-$SO_4(H_3O)_2$ S13

Figure S8. SXPD patterns and Rietveld refinement for MFM-300(Cr)-$SO_4(H_3O)_2$ S13

Table S1. Unit cell parameters for MFM-300(Cr)-$SO_4(H_3O)_2$ and MFM-300(Cr) S14

Figure S9. Nyquist plots for MFM-300(Cr). S15

Figure S10. An example of an equivalent circuit for fitting the proton conduction data for MFM-300(Cr). S15

Figure S11. An example of an equivalent circuit for fitting for MFM-300(Cr)-$SO_4(H_3O)_2$ S16

Figure S12. Proton conductivity for MFM-300(Cr) and MFM-300(Cr)-$SO_4(H_3O)_2$ S16

Table S2. Comparison of reported activation energies for MOFs S17

Figure S13. An example of HWHM fitting of QENS spectra of MFM-300(Cr)-$SO_4(H_3O)_2$ S18

Figure S14. Q^2-dependence of HWHM of QENS spectra and E_a for MFM-300(Cr) S19

Figure S15. Comparison of diffusion coefficients for MFM-300(Cr) and MFM-300(Cr)-$SO_4(H_3O)_2$ S20

3. Supplementary Notes S21

Figure S16. Arrhenius plots for MFM-300(Cr)-$SO_4(H_3O)_2$ under various RH conditions S21

Figure S17. Water vapor sorption isotherms for MFM-300(Cr) and MFM-300(Cr)-$SO_4(H_3O)_2$ at 25 °C..... S21

Figure S18. Proton conductivity and PXRD for MFM-300(Cr)-$SO_4(H_3O)_2$ stored for two years S22

Figure S19. NPD refinement and views of structure of MFM-300(Cr)-$SO_4(H_3O)_2$.......... S23

Figure S20. *In situ* FT-IR spectra for MFM-300(Cr)-$SO_4(H_3O)_2$ under various RH conditions. S24

Figure S21. MSD plots for MFM-300(Cr)-$SO_4(H_3O)_2$ from MD simulation.......... S25

4. Supplementary References S26

1. Materials and Methods

1.1 Synthesis

$CrCl_3 \cdot 6H_2O$ (>98%) was purchased from Acros Organics and chlorosulfonic acid (99%) from Sigma-Aldrich. Hydrochloric acid (37%), dichloromethane (DCM, >99%), methanol (>99%) and acetone (>99%) were purchased from Fisher Scientific. All chemicals were used as purchased without further purification. The ligand, biphenyl-3,3',5,5'-tetracarboxylic acid (H_4L), was synthesized by the reported method.[1]

MFM-300(Cr) was synthesized following our previously reported method.[2] In a typical synthesis, $CrCl_3 \cdot 6H_2O$ (283 mg, 1.06 mmol), H_4L (70 mg, 0.21 mmol), deionized water (10 mL) and HCl (1%, 1.5 mL) were mixed in a Teflon-lined steel autoclave, which was sealed and heated at 210 °C in an oven for 3 days. The resultant blue solid was isolated by centrifugation (9000 rpm, 5 min), washed with water and acetone twice, and dried at 50 °C to afford the product, Elemental analysis (%) for $[Cr_2(OH)_2(C_{16}H_8O_8)](H_2O)_{5.2}$: calc. 18.6% Cr, 3.7% H, 34.3% C, 0% Cl; found 17.8% Cr, 3.9% H, 33.6% C, 0% Cl.

Post-synthetic modification of MFM-300(Cr) (1.00 g) was carried out by stirring in DCM (30 mL) at 0 °C for 30 mins, followed by dropwise addition of DCM (10 mL) that contained chlorosulfonic acid (1.00 g). After 2h the solid product was separated through centrifugation, and washing with fresh DCM three times before drying at 50 °C to give MFM-300(Cr)-$SO_4(H_3O)_2$. Elemental analysis for $[Cr_2(OH)_2(C_{16}O_8H_6)](H_2SO_4)_{0.86}(H_2O)_{7.38}$: calc. 15.3% Cr, 3.6% H, 28.3% C, 4.0% S, 0% Cl; found 15.9% Cr, 3.3% H, 29.1% C, 3.5% S, 0% Cl. It was found that direct reaction with H_2SO_4 does not afford such high loadings of SO_4^{2-} compared to the use of $ClSO_3H$. $ClSO_3H$ is very sensitive to water and forms H_2SO_4 within the pores with volatile HCl being expelled. This can be expressed by the equation:

$$ClSO_3H + H_2O\ (g) \rightarrow H_2SO_4 + HCl\ (g).$$

1.2 General characterization

Elemental analyses were carried out on a FLASH 2000 elemental analyser. Scanning electron microscopy (SEM) images of MFM-300(Cr)-$SO_4(H_3O)_2$ were captured on a Zeiss Scanning Electron microscope with an EDS7636 SiLi dectector for EDX measurements. Infrared spectra were collected in the range of 4000–500 cm^{-1} by a Thermo Scientific Nicolet iS5 spectrometer with iD5 ATR attachment. ^1H NMR spectra were collected on a B400 Bruker Avance III 400 MHz. Samples were digested in 500 μL D_2O and 50 μL 35 wt% deuterium chloride at 120 °C overnight, and products were collected by centrifugation (10000 rpm, 5 min) and dissolved in 500 μL DMSO-d_6 for NMR analysis. Thermogravimetric analysis (TGA) was conducted under air flow (100 $mL \cdot min^{-1}$) at a heating rate of 10 $°C \cdot min^{-1}$ from room temperature to 800 °C through a Pyris 1 Thermogravimetric Analyser (Perkin Elmer). The exhaust gases and products produced by heating the sample at a rate of 10 $°C \cdot min^{-1}$ from room temperature to 600 °C were analysed by a Bruker Matrix MG5 FTIR spectrometer (resolution: 0.5 cm^{-1}) to detect SO_2. Powder X-ray diffraction (PXRD) patterns were measured over 2θ range of 5–50° by using a Panalytical X'Pert MPD diffractometer with Cu-Kα (λ = 1.54056

Å) at the working condition of 40 kV and 30 mA. Water vapor isotherms were collected on an Autosorb iQ Gas Sorption System at 298 K. Samples were activated at 150 °C for 12 h before measurements were undertaken.

1.3 Structure determination and refinements of synchrotron XPD data

The synchrotron X-ray powder diffraction (SXPD) experiments were carried out at Beamline I11 Diamond Light Source (Oxford, UK) using high-resolution synchrotron diffraction [λ = 0.826562(2) Å]. The sample powder was loaded into a 0.7 mm borosilicate glass capillary. High-resolution SXPD data were collected in the 2θ range 0–150° with 0.001° data steps using the multi-analyser crystal detectors at 25.0 °C. The SXPD patterns were refined using the Rietveld method in the TOPAS software.[3] Stephen fitting was applied to describe the diffraction peaks and their anisotropic broadening.[4] The scale factor and lattice parameters were allowed to refine for all the diffraction patterns. The refined structural parameters include the fractional coordinates (x, y, z) and isotropic displacement factors for all the atoms, and the site occupancy factors for guest molecules. The final stage of the Rietveld refinement involved soft restraints to the C–C bond lengths within the benzene rings. Rigid body refinement was applied to the guest molecules in the pore. The quality of the Rietveld refinements was assured with low weighted profile factors and well-fitted patterns with reasonable isotropic displacement factors within experimental errors.

1.4 Impedance measurements

Proton conductivity determination was based on impedance analysis performed on a Solatron SI 1260 Impedance/Gain phase Analyzer over a frequency range of 1–10^6 Hz with an amplitude of 100 mV and a DC rest voltage of 0 mV. The sample (~70 mg) was finely ground and pressed into a pellet of 8.00 mm in diameter and ~1 mm in thickness. The pellet was coated with silver paste on both sides to improve contact with the platinum electrodes in an electrochemical cell for proton conductivity measurement. Each measurement was carried out when the sample was maintained at a target temperature and relative humidity (RH) for 6 h. The temperature and RH were controlled by a Kambic KK-50 climatic chamber and monitored by a Rotronic HC2-C04 probe. Proton conductivity (σ, S cm^{-1}) was calculated by $\sigma = l/(R\cdot S)$, in which S and l are the cross-sectional area and thickness of the sample pellet respectively, and R is the resistance of the sample determined from the impedance plots by using ZView software. Activation energy (E_a) of proton conductivity (σ) was extracted from the data measured at various temperatures (99% RH) by using the Arrhenius equation: $\sigma = (\sigma_0/T)\cdot\exp[-E_a/(k\cdot T)]$, where σ_0, T and k are the pre-exponential factor, temperature and Boltzmann constant, respectively.

1.5 Quasi-elastic neutron scattering (QENS)

The proton dynamics in MFM-300(Cr)-$SO_4(H_3O)_2$ were probed on the neutron spectrometer (IRIS) at the ISIS Pulsed Neutron and Muon Source, Chilton, UK.[5] IRIS is a time-of-flight inverted-geometry neutron spectrometer with a single crystal array of pyrolytic graphite as an energy analyzer that makes use of Bragg

reflections close to backscattering geometry (Bragg angle θ_B = 175°) to analyze the energy of scattered neutrons. The detector array that counts those scattered neutrons covers a scattering angle (2θ) range of 27–158° yielding a wave vector (Q) range of 0.4–1.8 $Å^{-1}$. In this measurement, IRIS was performed in the PG(002) configuration that offers an energy resolution of 17 μeV and an energy window (ħω) of -0.5–0.5 meV. The sample (~1.2 g) was packed into an annular aluminum container with an appropriate thickness (0.5 mm) to minimize multi-scattering effects. Data collection was made at every 10 K in the temperature range of 8–353 K, with long scanning (6 h) at 8 K (for resolution), and then 233, 273 K. The obtained spectra were fitted by a delta function and a single Lorentzian function, convoluted by the resolution function. The spectrum of corresponding samples measured at base temperature was used as a resolution function, assuming that no motions are expected to occur at such low temperature. The fitting also involved a term of linear background.[6] The half-width at half-maximum (HWHM) of obtained spectra were fitted by jump diffusion models shown below. Equation S1–S4 are Fick Model, Chudley-Elliot Model, Singwi-Sjölander Model, and Hall-Ross Model,[7] respectively, where Γ is the HWHM of QENS spectra, and l, τ and D are the jump length, relaxation time and diffusion coefficient of the diffusing particles measured, respectively.

$$\Gamma(Q) = \hbar \cdot D \cdot Q^2 \qquad \text{(S1)}$$

$$\Gamma(Q) = \frac{\hbar}{\tau}\left[1 - \frac{\sin(Q \cdot l)}{Q \cdot l}\right] \qquad \text{(S2)}$$

$$\Gamma(Q) = \frac{\hbar}{\tau}\frac{Q^2 \cdot l^2}{1 + Q^2 \cdot l^2} \qquad \text{(S3)}$$

$$\Gamma(Q) = \frac{\hbar}{\tau}\left(1 - e^{-Q^2 l^2/6}\right) \qquad \text{(S4)}$$

1.6 Neutron powder diffraction (NPD) experiment and refinement

Structural determination of the binding positions of sulfuric acid within MFM-300(Cr)-$SO_4(H_3O)_2$ were conducted on WISH, a long-wavelength powder and single crystal neutron diffractometer at the ISIS neutron and muon facility at Rutherford Appleton Laboratory (UK). The instrument incorporates a solid methane moderator, providing a high flux of cold neutrons with a large bandwidth, which is transported to the sample via an elliptical guide. The divergence jaws of WISH allow tuning of the resolution according to the need of the experiment; in this case, it was setup in high-resolution mode. The WISH detectors are 1 m long, 8 mm diameter pixelated ^{3}He tubes positioned at 2.2 m from the sample and arranged on a cylindrical locus covering a 2θ scattering angle of 10–170°. To reduce the background from the sample environment, WISH is equipped with an oscillating radial collimator that defines a cylinder of radius of approximately 22 mm diameter at 90° scattering. The MOF sample was loaded into a cylindrical vanadium sample container with an indium vacuum seal. NPD data was collected at 298 K.

Rietveld refinements of the NPD patterns of MFM-300(Cr)-$SO_4(H_3O)_2$ were performed using the TOPAS software package. In this treatment the guest molecules are treated as rigid bodies. The centres of mass,

orientations, and occupancies of the adsorbate were first refined, followed by full profile Rietveld refinement, including the positions of metals and linkers together with their corresponding lattice parameters, resulting in satisfactory R-factors. The final refinements on all parameters including fractional coordinates, thermal parameters, occupancies for both host lattice and adsorbate molecules, and background/profile coefficients yielded very good agreement factors.

1.7 *In situ* infrared spectroscopy

In situ DRIFTS measurements were carried out offline with a thermal IR (globar) source at beamline B22 at Diamond Light Source using a Pike DiffusIR DRIFTS cell and HTV Environmental Chamber with a ZnSe window in combination with a Bruker Vertex 80V FTIR equipped with a mid-infrared LN2-cooled MCT (Mercury Cadmium Telluride) detector. The gas environment was controlled *via* a custom mass flow controller set-up in combination with a custom bubbler system. Samples were prepared by diluting the MOFs in KBr (5 wt. %) before being ground to a fine powder with a pestle and mortar for 5 minutes. Desolvated samples were generated *in situ via* activation at 523 K under a flow of dry N_2 at 100 $cm^3 \cdot min^{-1}$ until the solvent peaks had disappeared from the spectrum. Samples were then cooled to 298 K and exposed to incrementally to increasing relative humidities by passing dry N_2 through distilled water in a custom bubbler system.

1.8 Molecular dynamics (MD) simulation

MD simulations were performed using the Vienna Ab initio Simulation Package (VASP).[8] The calculation used Projector Augmented Wave (PAW) method[9,10] to describe the effects of core electrons, and Perdew-Burke-Ernzerhof (PBE)[11] implementation of the Generalized Gradient Approximation (GGA) for the exchange-correlation functional. Energy cutoff was 500 eV for the plane-wave basis of the valence electrons. The lattice parameters and atomic coordinates measured by NPD in this work were used as the initial structure. The total energy tolerance for electronic energy minimization was 10^{-4} eV. The optB86b-vdW functional[12] for dispersion corrections was applied. The ab initio MD simulations were performed at 500 K under NVT ensemble (an elevated temperature was used to accelerate the dynamics so that diffusive events can be observed within the timescale of the simulation). The time step was 0.5 fs, and a total of 72000 steps were simulated.

2. Supplementary Figures and Tables

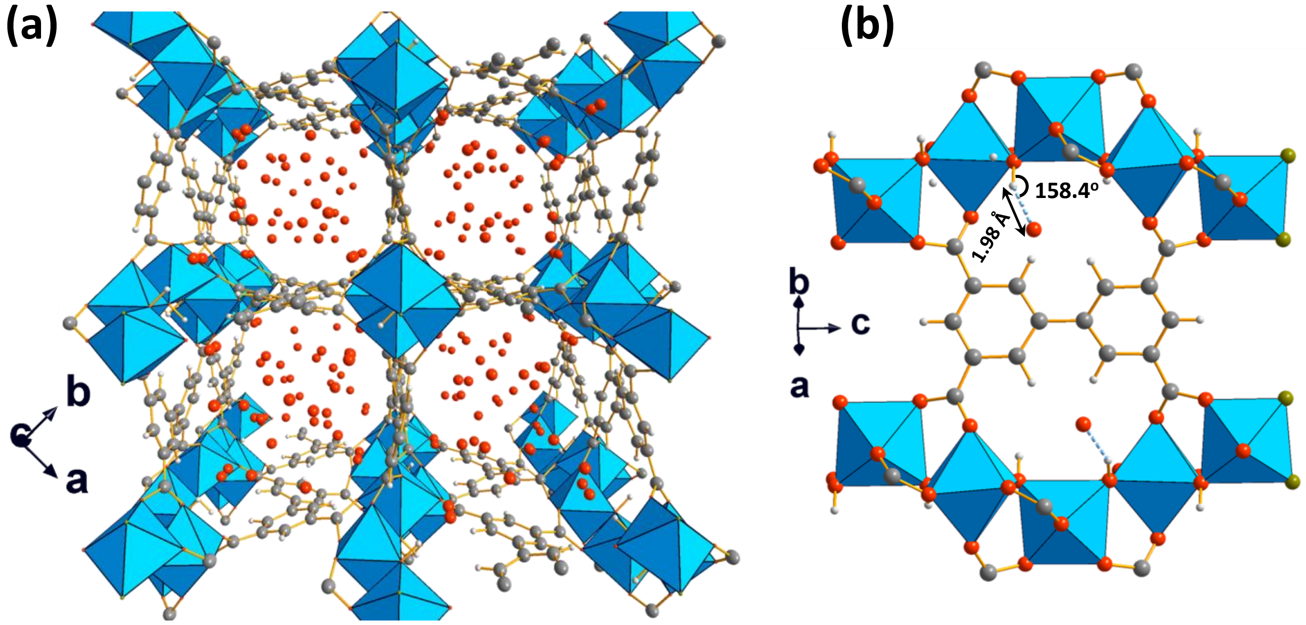

Figure S1. (a) View of the 3D framework structure of MFM-300(Cr) along the crystallographic *c*-axis. Molecules of disordered water are shown in the channels. (b) View of the structure of MFM-300(Cr) along the (110) axis.

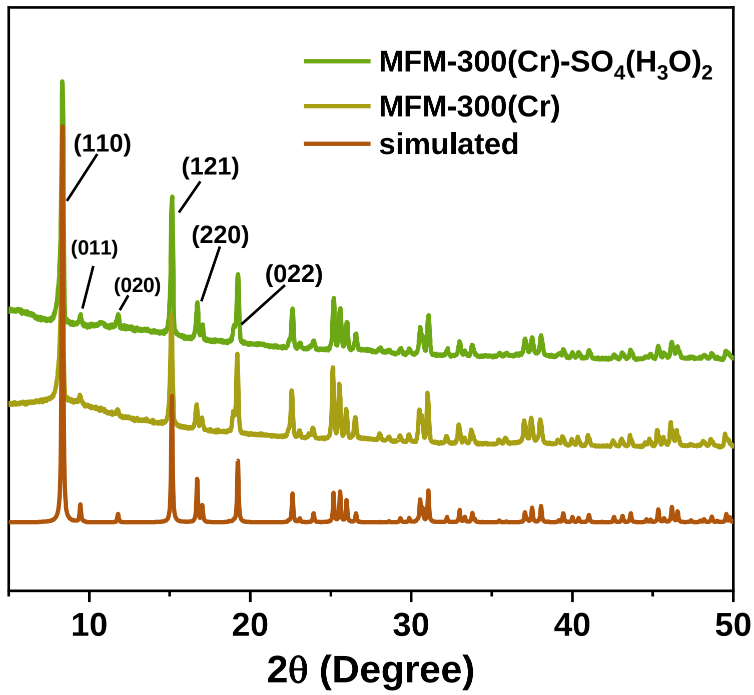

Figure S2. PXRD patterns for simulated MFM-300(Cr), as-synthesized MFM-300(Cr) and MFM-300(Cr)-$SO_4(H_3O)_2$.

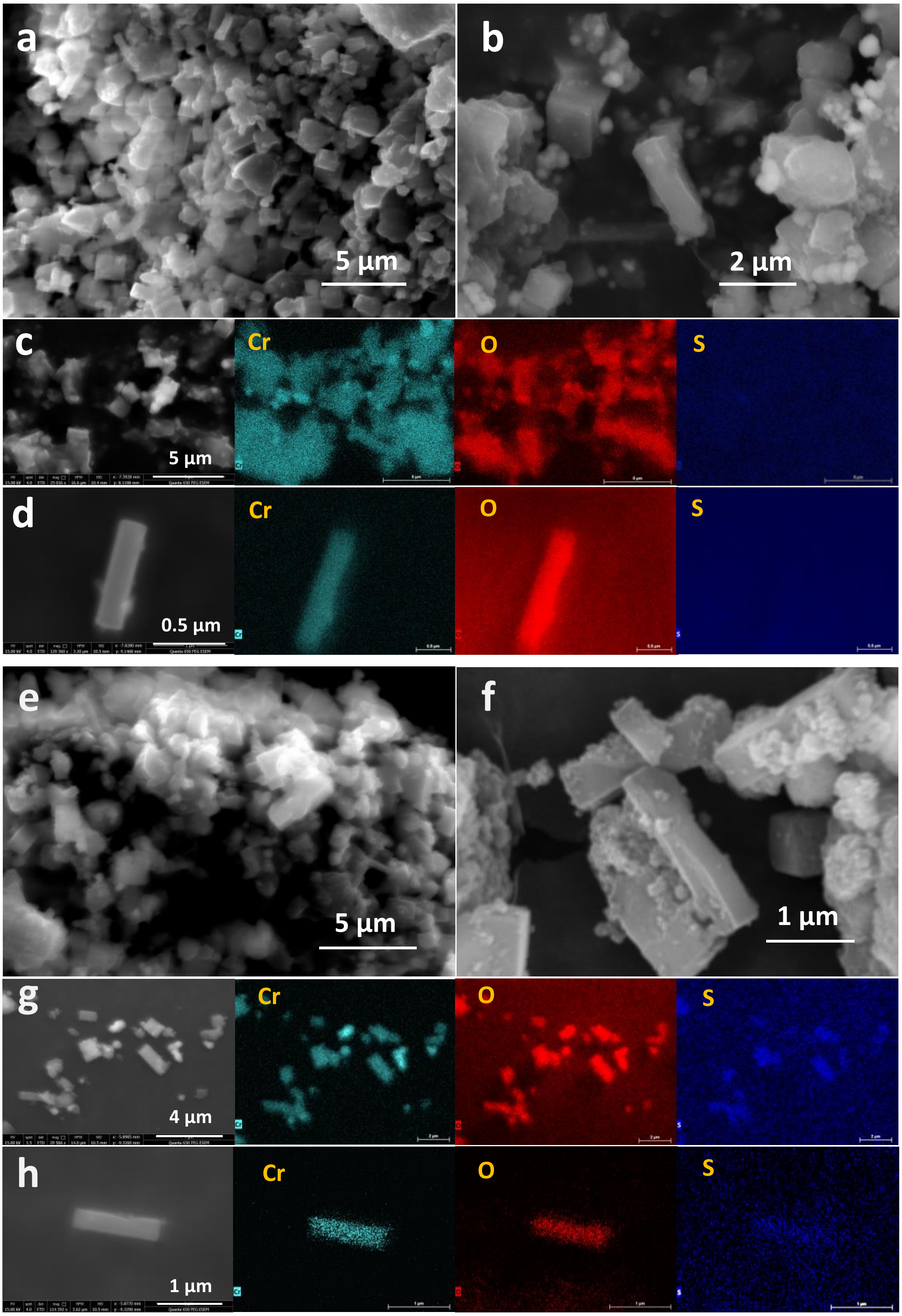

Figure S3. SEM images for (a–d) MFM-300(Cr) and (e–h) MFM-300(Cr)-$SO_4(H_3O)_2$ and the corresponding EDX mapping for chromium, oxygen and sulfur.

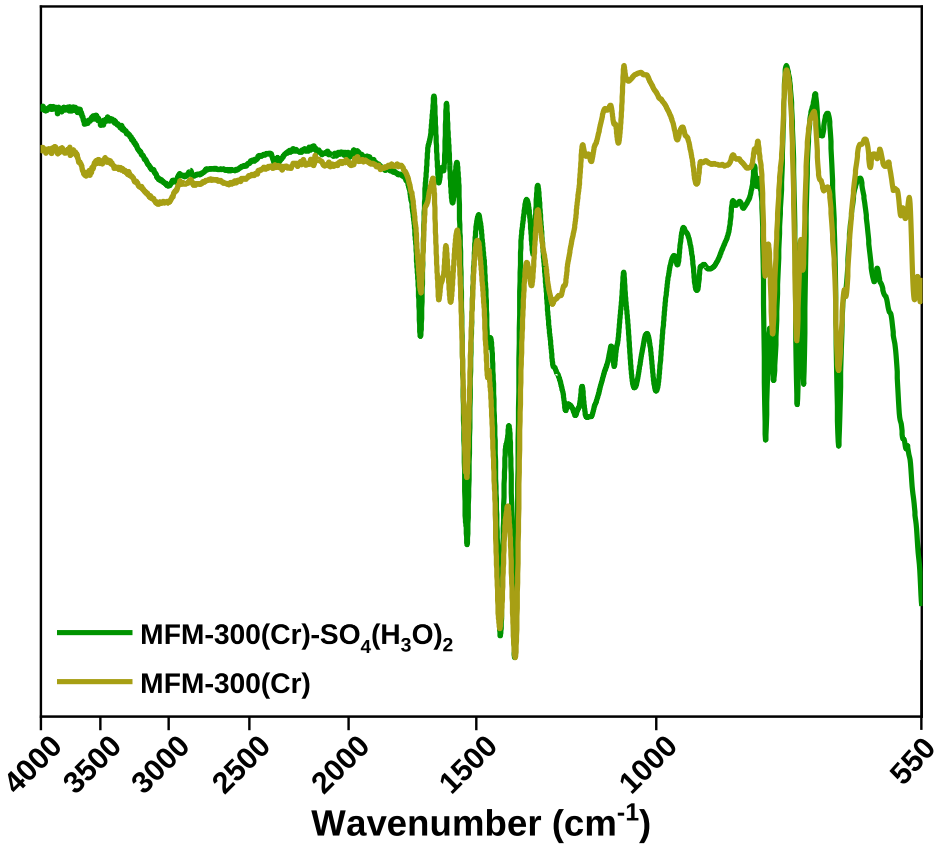

Figure S4. Infrared spectra of MFM-300(Cr) and MFM-300(Cr)-$SO_4(H_3O)_2$.

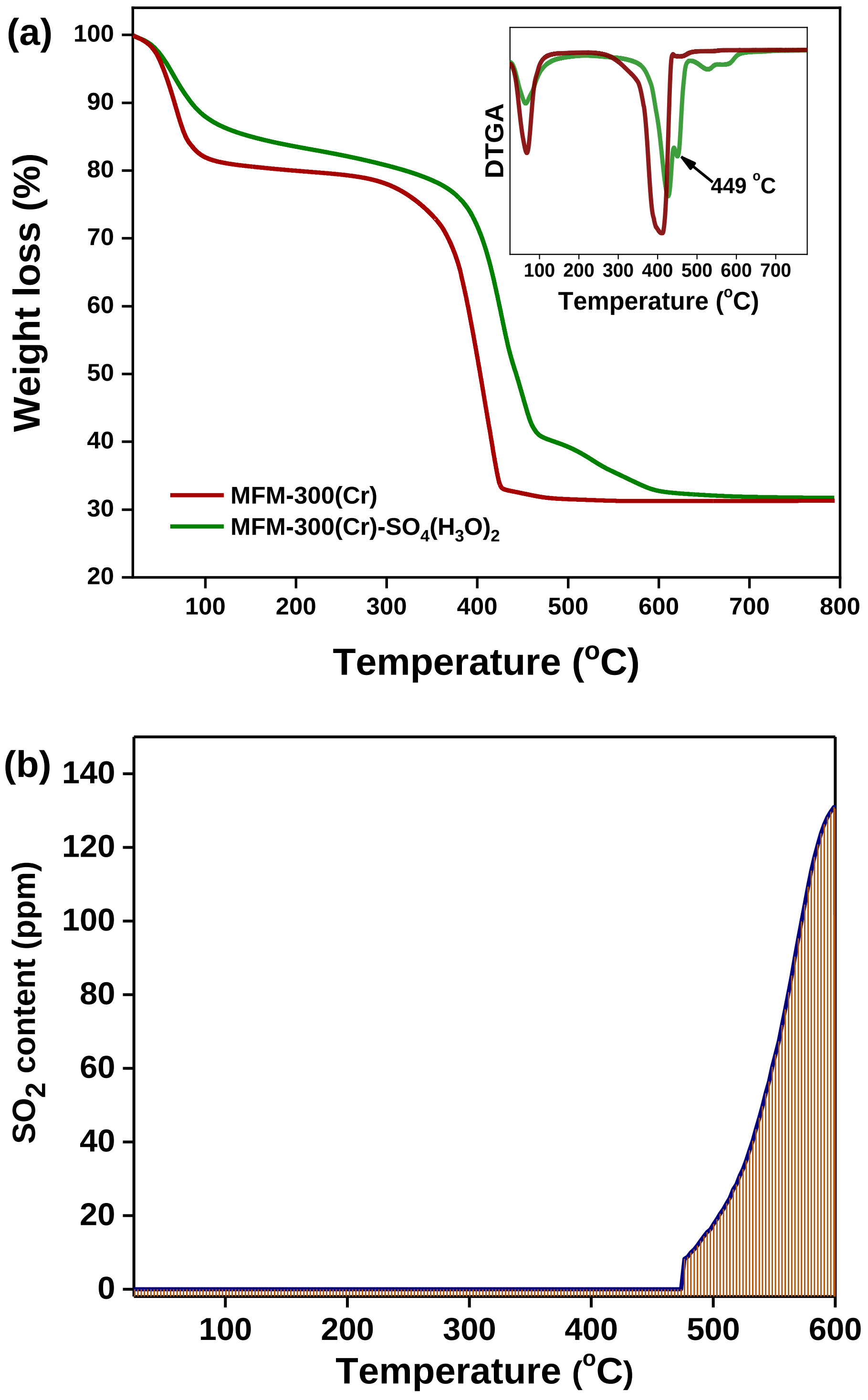

Figure S5. (a) TGA profiles of MFM-300(Cr) and MFM-300(Cr)-$SO_4(H_3O)_2$. The inset is the derivative TGA plots for these two materials. The ratio of Cr to S was calculated to be 1:0.34, consistent with the results from elemental analysis (1:0.30). (b) Monitoring of SO_2 emission using an infrared detector on heating of MFM-300(Cr)-$SO_4(H_3O)_2$. No SO_2 was detected on heating MFM-300(Cr).

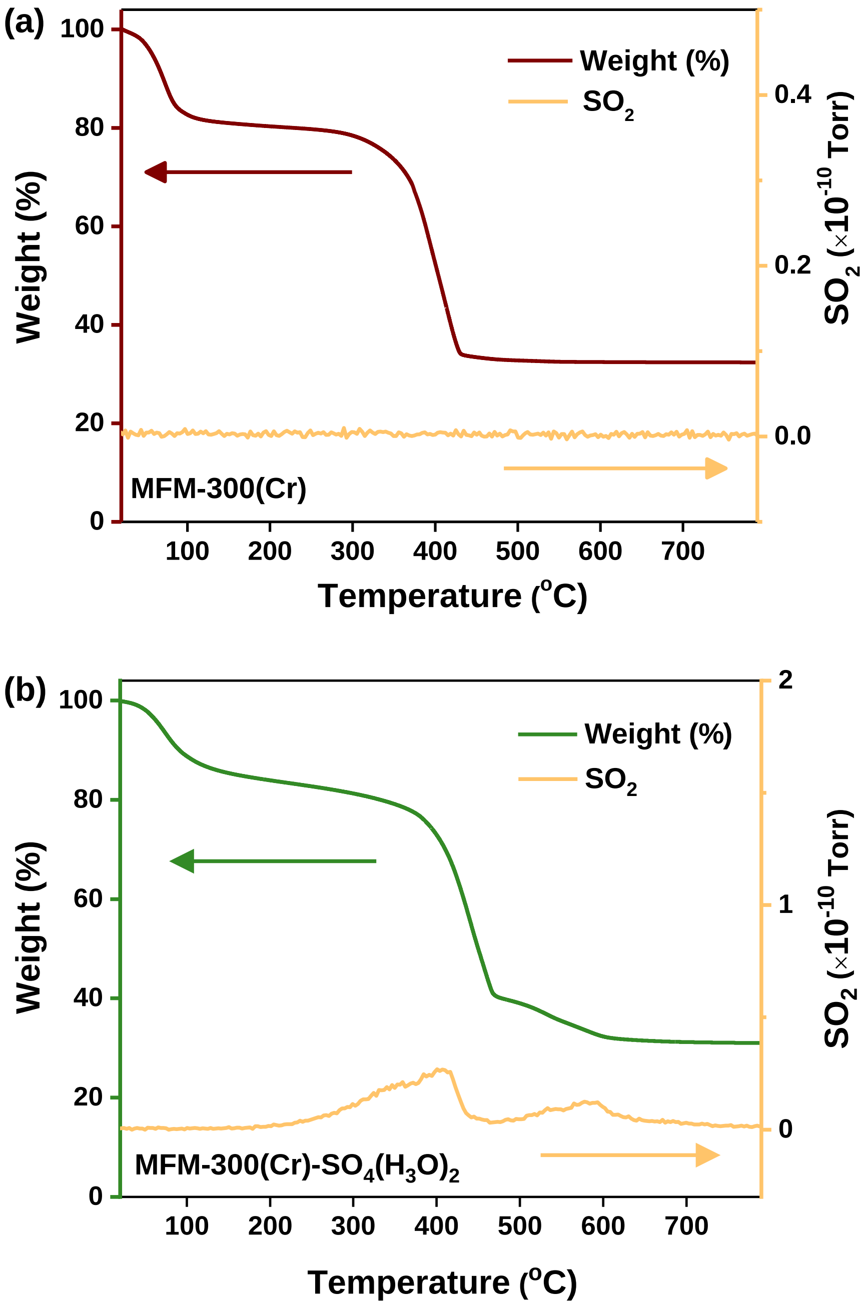

Figure S6. (a) TGA profiles and SO_2 monitoring on heating of MFM-300(Cr). (b) TGA profiles and SO_2 monitoring on heating of MFM-300(Cr)-$SO_4(H_3O)_2$.

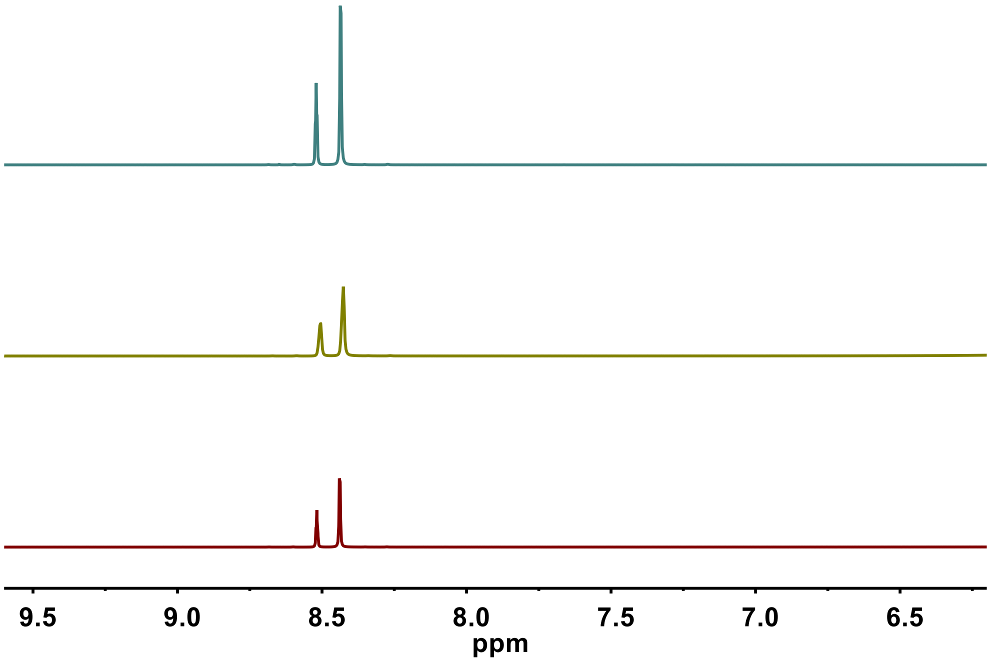

Figure S7. ^{1}H NMR spectrum in deuterated dimethyl sulfoxide (DMSO-d_6) of H_4L (blue, top), digested MFM-300(Cr) (yellow, middle) and digested MFM-300(Cr)-$SO_4(H_3O)_2$ (maroon, bottom).

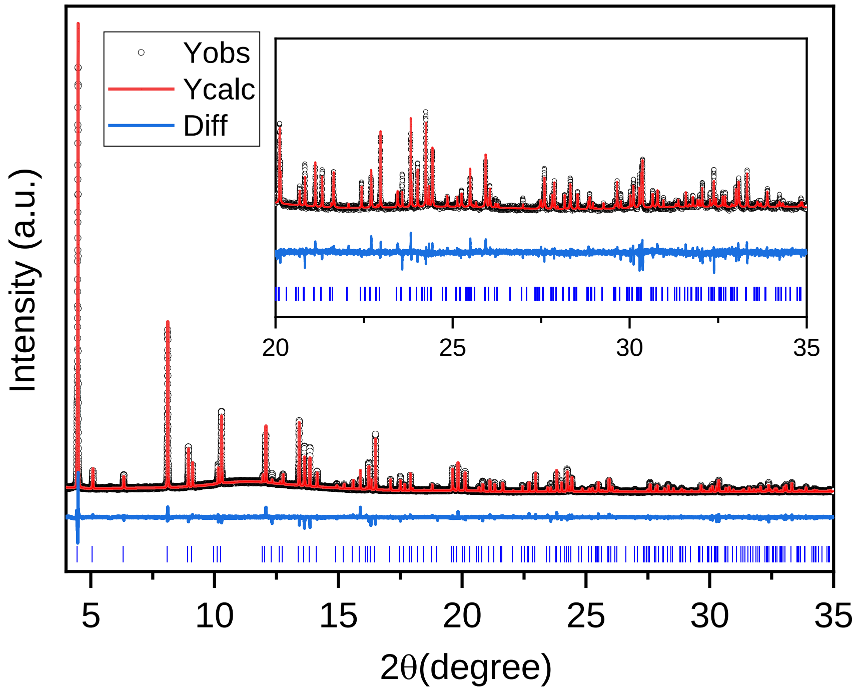

Figure S8. Comparison of the experimental data, Rietveld refinement and their difference for SXPD patterns of MFM-300(Cr)-$SO_4(H_3O)_2$. The inset shows an enlarged view of the region from 20° to 35°.

Table S1. Unit cell parameters from SXPD and NPD study for MFM-300(Cr)-$SO_4(H_3O)_2$ and MFM-300(Cr).

	**MFM-300(Cr)**	**MFM-300(Cr)-$SO_4(H_3O)_2$**	**MFM-300(Cr)-$SO_4(H_3O)_2$**
**Formula**	$[Cr_2(OH)_2(C_{16}O_8H_6)](H_2O)_{5.20}$	$[Cr_2(OH)_2(C_{16}O_8H_6)](H_2SO_4)_{1.00}(H_2O)_{5.88}$	$[Cr_2(OH)_2(C_{16}O_8H_6)](H_2SO_4)_{0.86}(H_2O)_{7.38}$
**Crystal system**	Tetragonal	Tetragonal	Tetragonal
**Space group**	$I4_122$	$I4_122$	$I4_122$
**a (Å)**	15.03770(5)	14.787(4)	15.04657(8)
**b (Å)**	15.03770(5)	14.787(4)	15.04657(8)
**c (Å)**	11.97032(5)	12.033(3)	11.98779(7)
**α (°)**	90	90	90
**β (°)**	90	90	90
**γ (°)**	90	90	90
**Cell volume (Å^3)**	2706.88(2)	2631(2)	2714.03(3)
***ρ* (g/cm^3)**	1.469	1.770	1.625
**Radiation type**	X-ray, Synchrotron	Neutron, TOF	X-ray, Synchrotron
**θ range (°)**	4–50°	N/A	4–50°
**Method**	Rietveld	Rietveld	Rietveld
**R_{wp}(%)**	8.58	0.88	7.57
**Goodness-of-fit**	2.25	2.99	1.90
**R_{bragg}(%)**	6.35	0.48	5.70
**R_{exp}(%)**	3.81	0.29	3.98
**CCDC number**	2164624	2164625	2164626

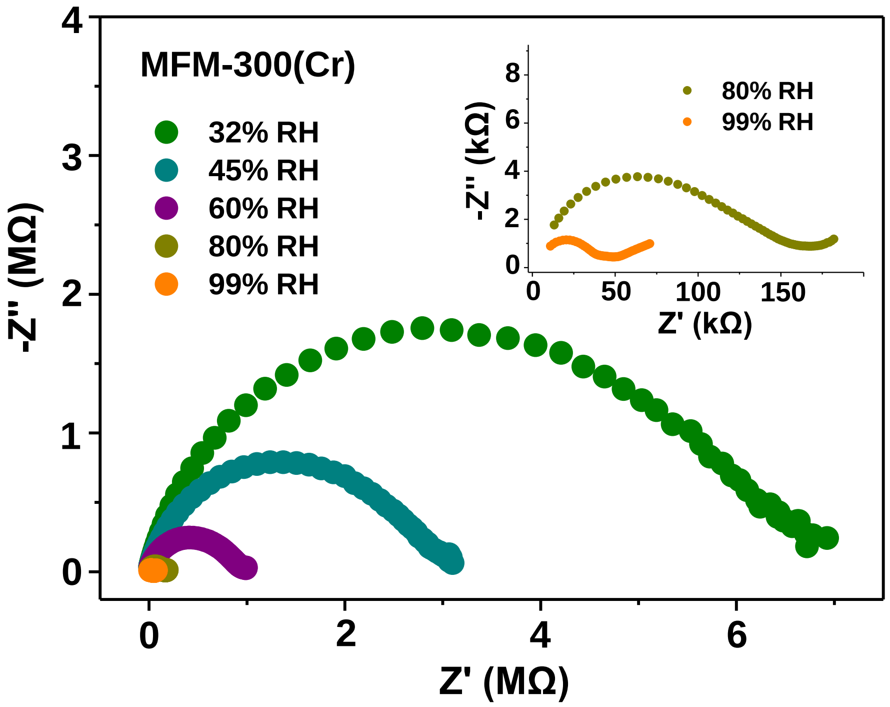

Figure S9. Nyquist plots for MFM-300(Cr) at room temperature. The inset shows an enlarged view of the high RH results for MFM-300(Cr).

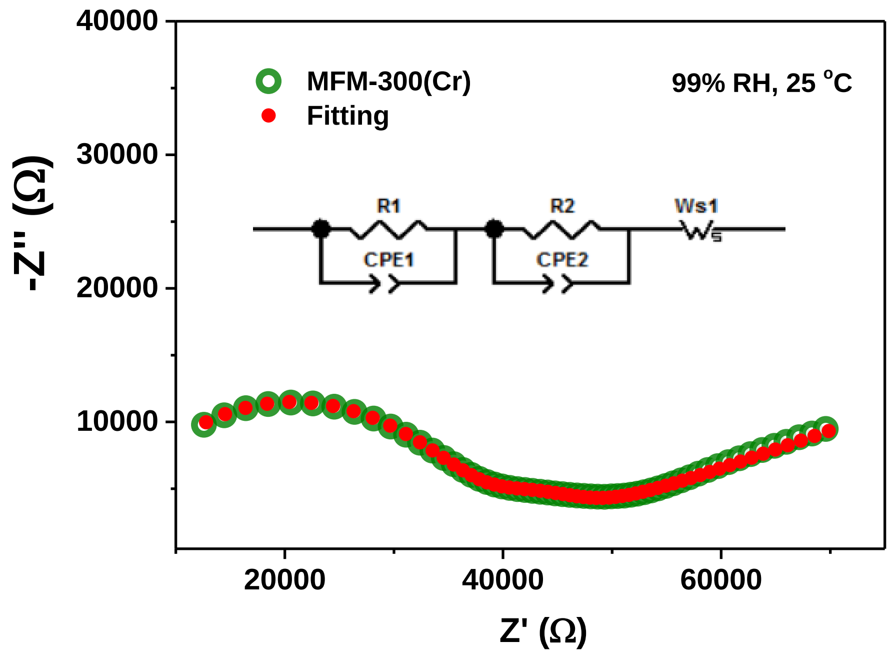

Figure S10. An example of an equivalent circuit for fitting the experimental proton conductivity data at room temperature and 99% RH for MFM-300(Cr).

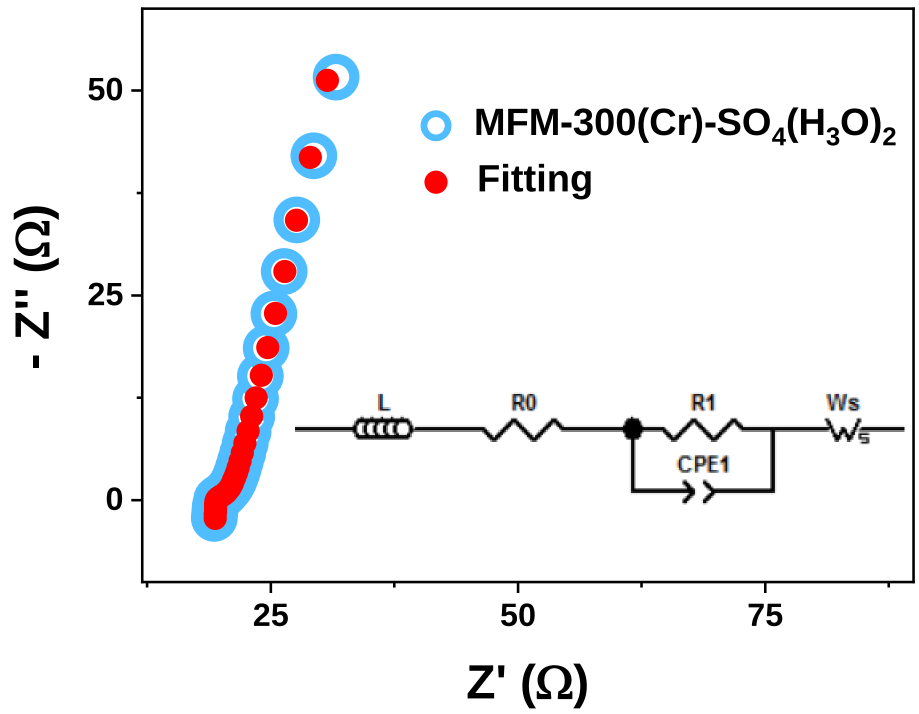

Figure S11. An example of an equivalent circuit for fitting the proton conductivity data at room temperature and 99% RH for MFM-300(Cr)-$SO_4(H_3O)_2$.

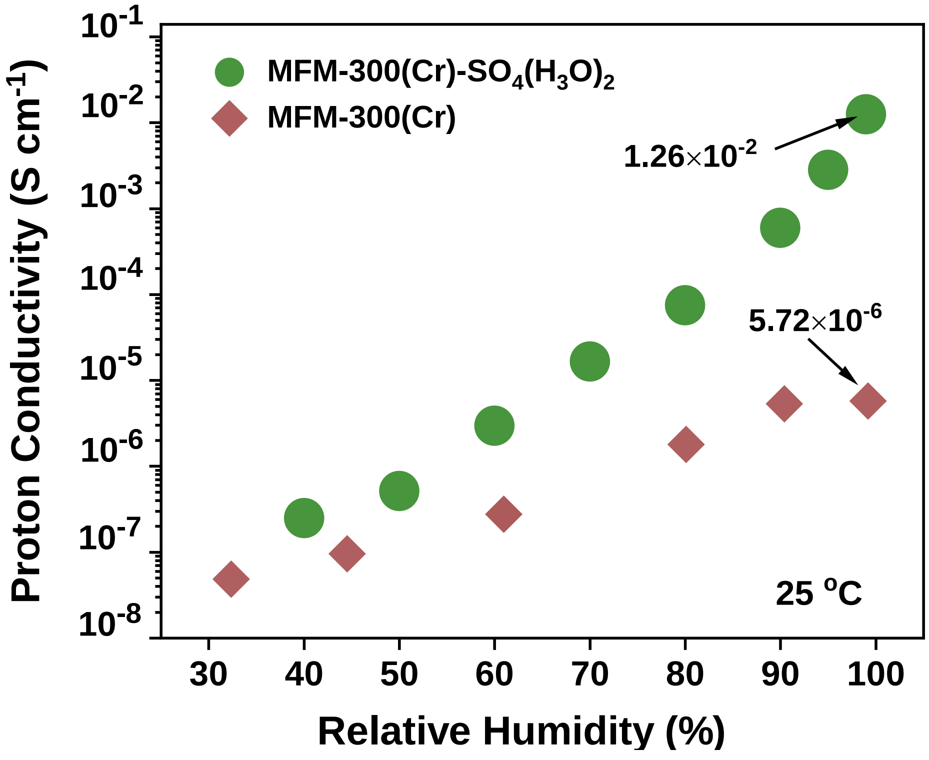

Figure S12. Proton conductivity of MFM-300(Cr) and MFM-300(Cr)-$SO_4(H_3O)_2$ under various RH at room temperature.

Table S2. Comparison of reported activation energies for MOFs with a proton conductivity of approximately 10^{-2} S·cm^{-1}.

**Materials**	**Ea / eV**	**σ / x 10^{-2} S·cm^{-1}**	**References**
MFM-300(Cr)-$SO_4(H_3O)_2$	0.04	1.26	This work
BUT-8(Cr)A	0.11	12.7	[13]
H_2SO4@MFM-555(Ho)	0.12	1	[14]
$[(Me_2NH_2)_3(SO_4)]_2[Zn_2(ox)_3]$	0.13	4.2	[15]
PCMOF2$^1/_2$(Pz)	0.16	11	[16]
TfOH@MIL-101	0.2	8	[17]
PCMOF2$^1/_2$	0.21	2.1	[18]
MIP-202(Zr)	0.22	1.1	[19]
$[Pt(dach)(bpy)Br]_4(SO_4)_4$	0.22	1.7	[20]
Nafion	0.22	5	[21]
PCMOF2$^1/_2$(Tz)	0.22	12	[16]
MIL-101-SO_3H	0.23	1.16	[13]
UiO-66-SO_3H	0.25	0.19	[22]
H_3PO_4@MIL-101	0.25	1	[23]
MFM-512	0.32	0.29	[24]
UiO-66-$(SO_3H)_2$	0.32	8.4	[25]
PCMOF10	0.4	3.55	[26]
MFM-500(Ni)	0.43	0.045	[27]
Im-Fe-MOF	0.44	1.21	[28]
$(NH_4)_2(adp)[Zn_2(ox)_3]\cdot 3H_2O$	0.63	0.8	[29]
CPM-103a	0.66	0.23	[30]

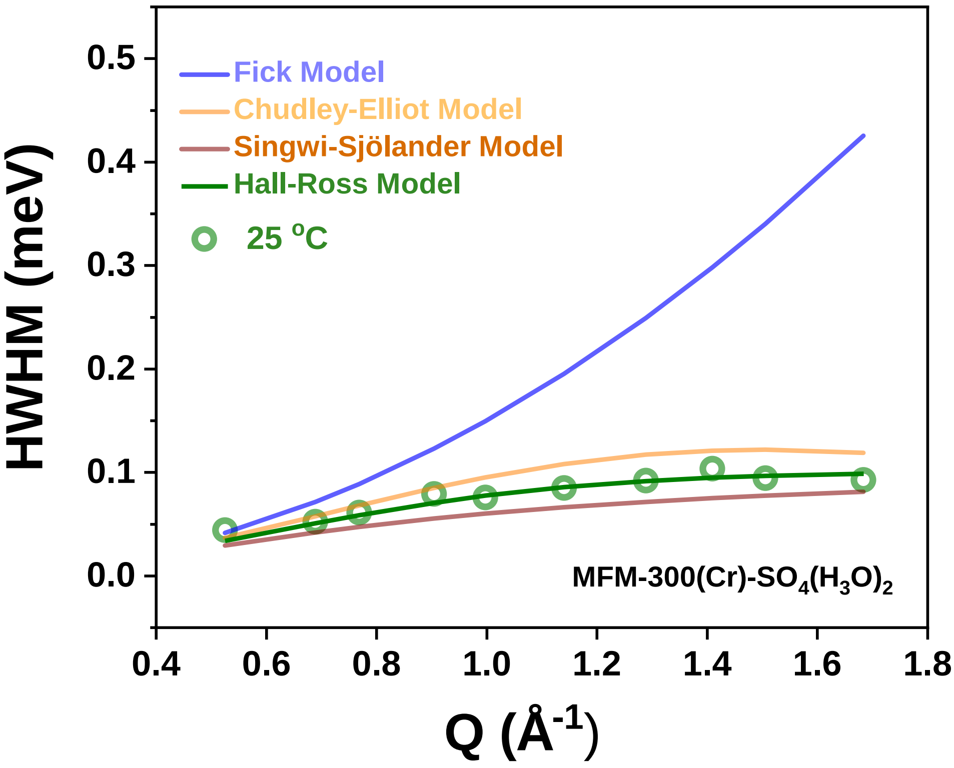

Figure S13. An example of the fitting of half-width at half-maximum (HWHM) from QENS spectra for MFM-300(Cr)-$SO_4(H_3O)_2$ using the relevant models shown above. (Jump length: 3 Å; relaxation time: 10 ps.)

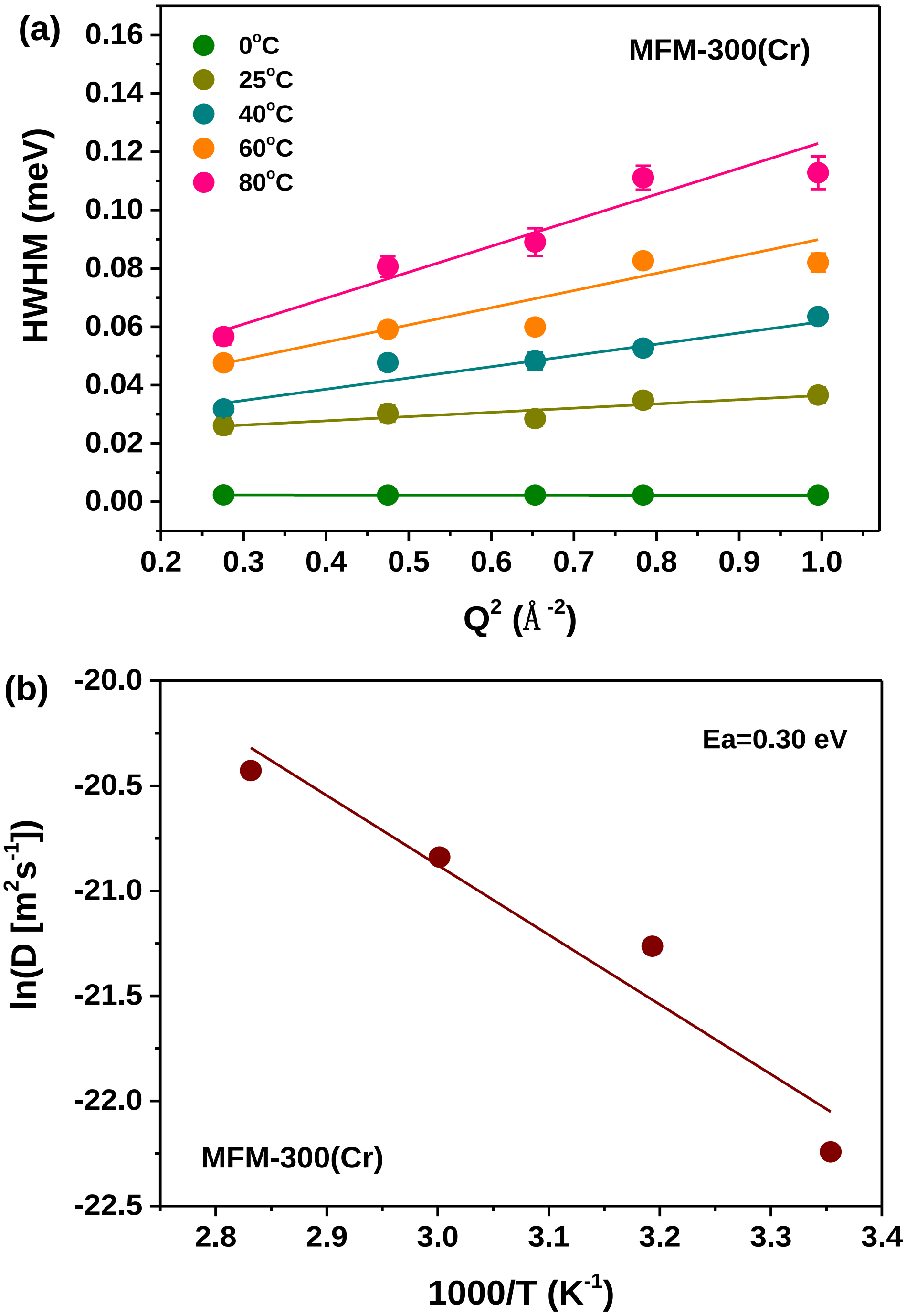

Figure S14. (a) Q^2-dependence of HWHM in the low Q region in the QENS spectra of MFM-300(Cr). (b) The Arrhenius plot of the diffusion coefficient derived from HWHM analysis for MFM-300(Cr). Note that the fitting of HWHM in the low Q region was made using HWHM = $\hbar DQ^2$ due to the noisy data collected in the high Q region for MFM-300(Cr).[31] For consistency, the same treatment was performed for MFM-300(Cr)-$SO_4(H_3O)_2$, showing similar results (E_a = 0.06 eV) to those calculated from the Hall-Ross model (E_a = 0.05 eV). It should be noted that the self-diffusion coefficient at 0 °C was excluded during Arrhenius fitting for MFM-300(Cr), given that the motions of protons in MFM-300(Cr) were too slow to be detected at 0 °C by IRIS (resolution: 0.17 μeV).

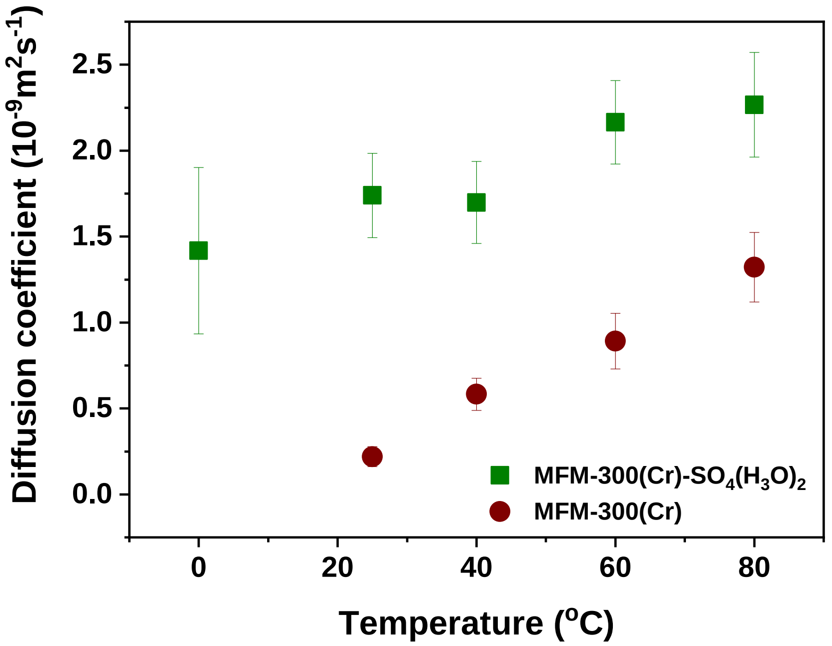

Figure S15. Comparison of the diffusion coefficient of MFM-300(Cr) and MFM-300(Cr)-$SO_4(H_3O)_2$ from 0~80 °C. Note that the diffusion coefficient for MFM-300(Cr) at 0 °C was excluded given that the motions of protons in MFM-300(Cr) were too slow to be detected at 0 °C by IRIS (resolution: 0.17 μeV).

3. Supplementary Notes

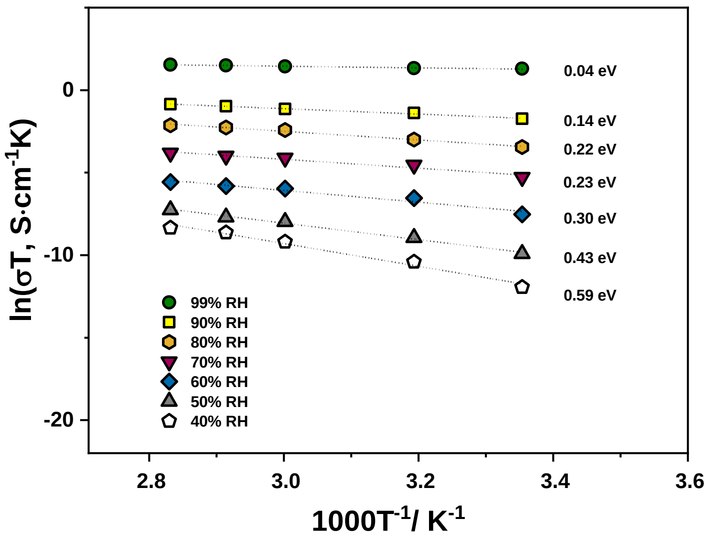

Figure S16. *Arrhenius* plots for the proton conductivity of MFM-300(Cr)-$SO_4(H_3O)_2$ under various RH conditions.

The activation energy of MFM-300(Cr)-$SO_4(H_3O)_2$ rises gradually with decreasing RH. When the humidity is lower than ~50% RH, the activation energy is higher than 0.4 eV, indicating that proton conduction is changed from the Grötthuss mechanism to the Vehicular mechanism. Thus, hopping through hydrogen-bonding networks is the dominant mechanism for long-range proton transfer in this material under >50% RH conditions. The guest water molecules play an important role in assembling the efficient hydrogen-bonding networks and achieving the observed low activation energy in this system.

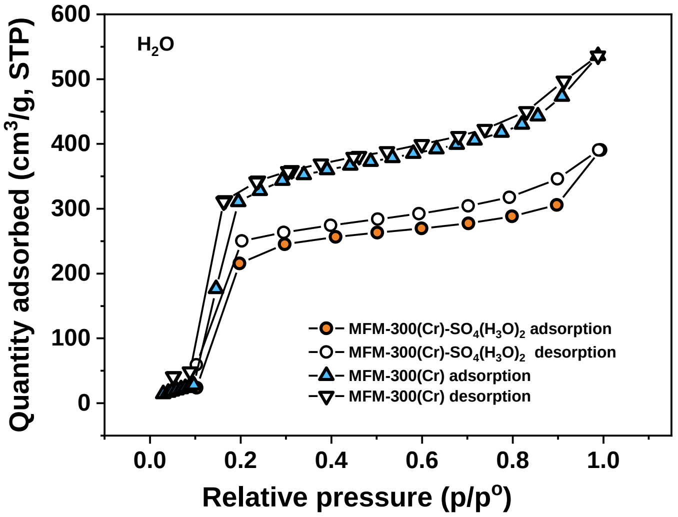

Figure S17. Water vapor sorption isotherms of MFM-300(Cr) and MFM-300(Cr)-$SO_4(H_3O)_2$ at 25 °C. Closed and open symbols denote the adsorption and desorption process, respectively. MFM-300(Cr)-$SO_4(H_3O)_2$ shows lower uptake than MFM-300(Cr) (17.4 and 23.9 mmol g^{-1}, respectively), consistent with the presence of $SO_4(H_3O)_2$ species that partially block the pores of the former.

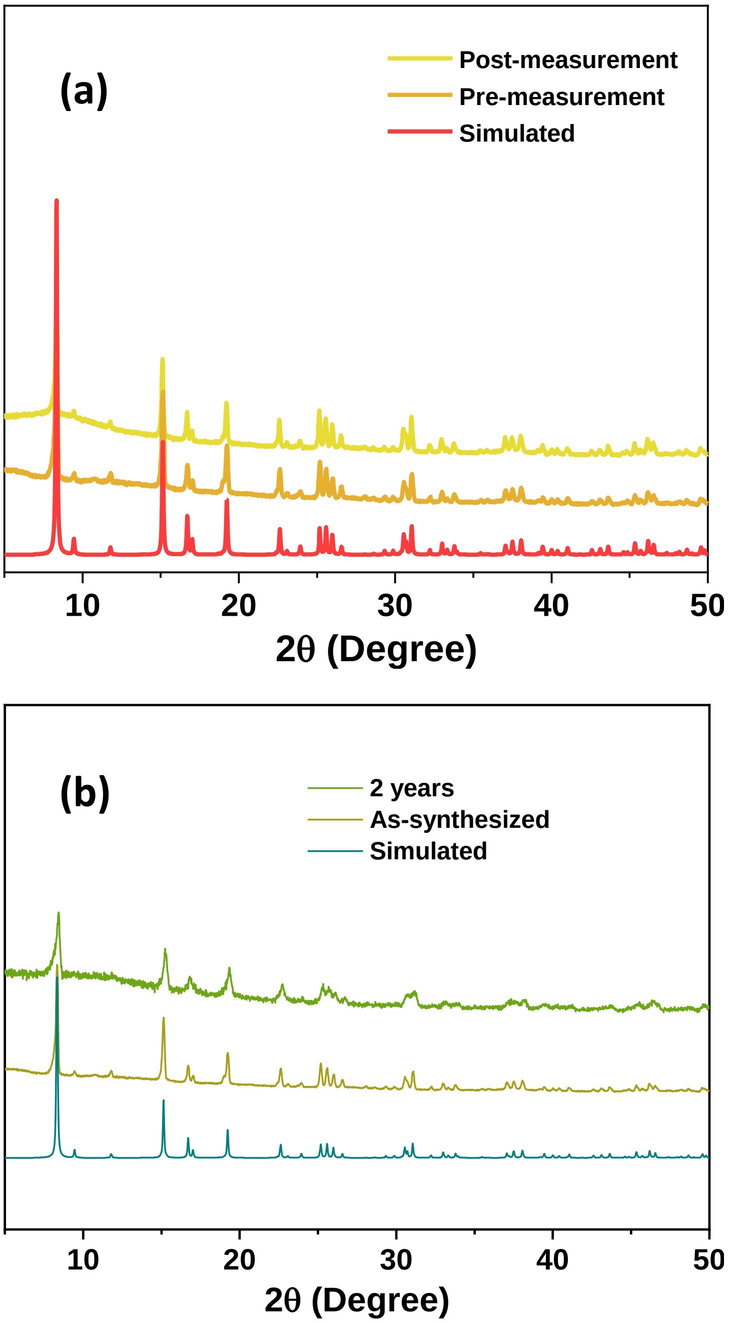

Figure S18. (a) PXRD patterns of MFM-300(Cr) (simulated), and MFM-300(Cr)-$SO_4(H_3O)_2$ before and after three cycles of impedance measurements. (b) PXRD patterns for as-synthesized MFM-300(Cr)-$SO_4(H_3O)_2$ and MFM-300(Cr)-$SO_4(H_3O)_2$ that has been stored under ambient conditions for 2 years.

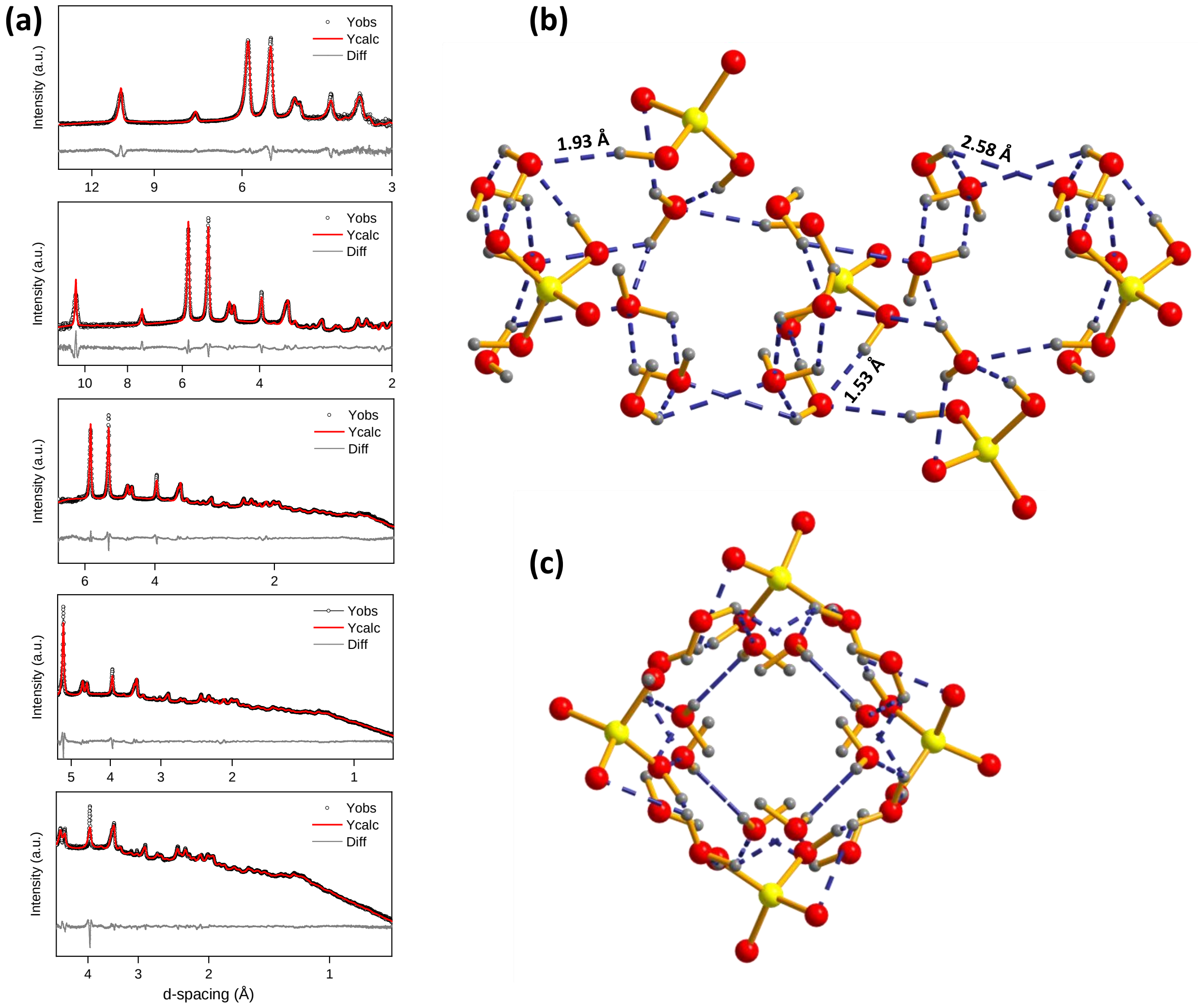

Figure S19. (a) Rietveld refinement of NPD data for MFM-300(Cr)-$SO_4(H_3O)_2$ (observed profile, black; calculated, red; difference, grey). The resulting crystallographic information is listed in Table S1. (b) View of hydrogen-bonding networks in 1D channels (b) along the *a*-axis and (c) the *c*-axis based on the results of NPD refinements (oxygen, red; sulfur, yellow; hydrogen, grey). Dashed lines illustrate potential pathways for proton transport, where hydrogen-bonding networks can be constructed. The structural model obtained from NPD is entirely consistent with that obtained from the PXRD refinements.

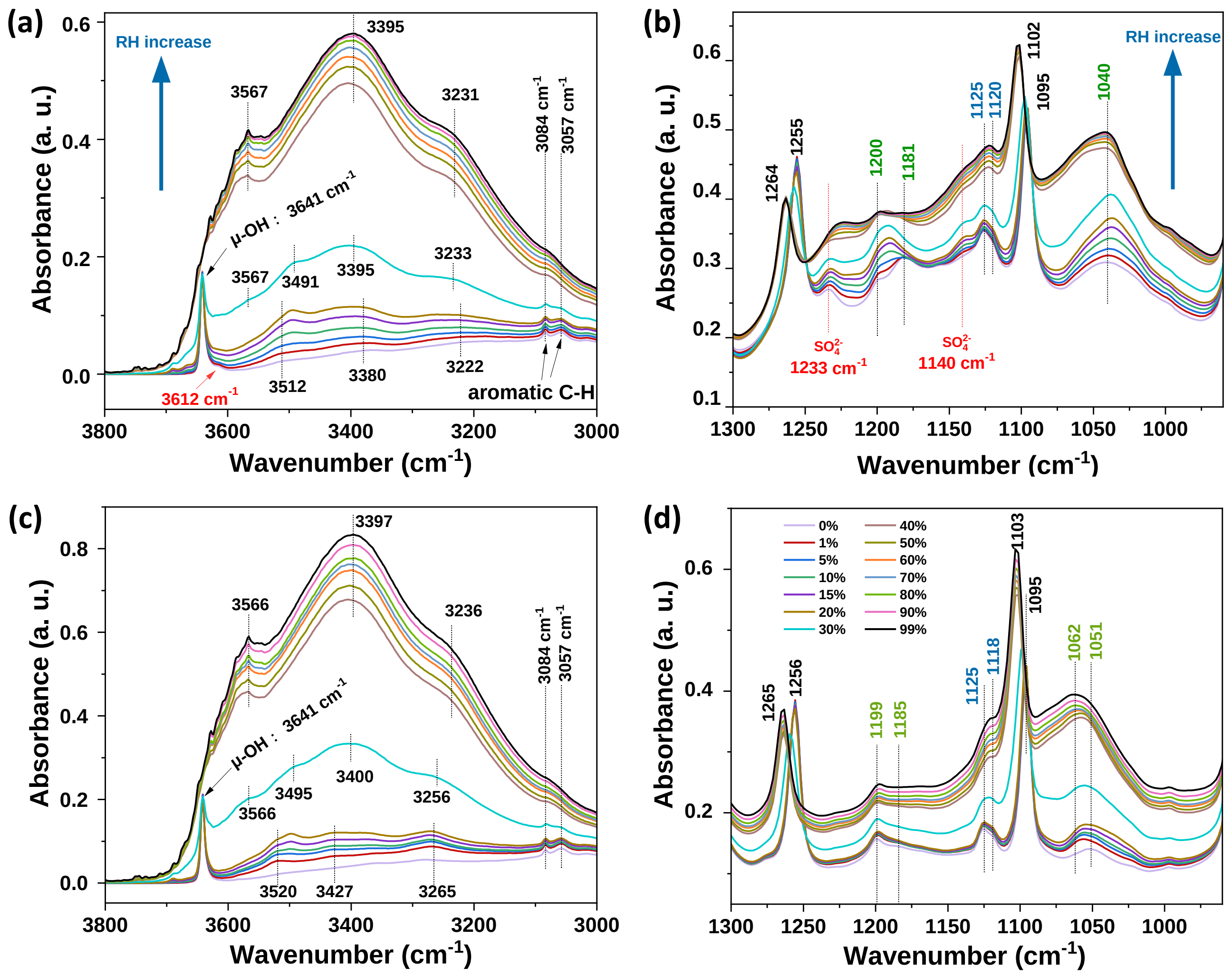

Figure S20. FT-IR spectra over (a) 3800–3000 cm^{-1} and (b) 1300–960 cm^{-1} for MFM-300(Cr)-$SO_4(H_3O)_2$ as a function of RH between 0–99%. FT-IR spectra over (c) 3800–3000 cm^{-1} and (d) 1300–960 cm^{-1} for MFM-300(Cr) as a function of RH between 0–99%. Color scheme for (a)–(c) is same as that given in (d).

Comparing Figure S20b with S20d, additional bands, labelled in red, are observed for MFM-300(Cr)-$SO_4(H_3O)_2$ at 1233 and 1140 cm^{-1}, resulting from asymmetric and symmetric ν(S=O) stretching modes. This suggests the presence of sulfate species in MFM-300(Cr)-$SO_4(H_3O)_2$. The sulfate species also influence the bands in the region of 1300–1000 cm^{-1} and the region of the O-H group at 3800–3000 cm^{-1} upon increasing RH. Interestingly, the spectra in Figure S20a and S20c reveal interaction between water molecules and the organic linker with two peaks from the aromatic C-H bonds (3084 and 3057 cm^{-1}) broadening due to hydrogen bonding as the humidity rises from 0% to 99% RH. More importantly, MFM-300(Cr)-$SO_4(H_3O)_2$ shows an additional peak at 3612 cm^{-1} (Figure S20a), consistent with the interaction of the bridging O-H groups and the confined SO_4^{2-} species within the pore (the ratio of SO_4^{2-}/-OH is *ca.* 1:2). This causes a red shift of the υ(O-H) stretching mode from 3641 cm^{-1} to 3612cm^{-1}, consistent with a previous report,[32] while the υ(O-H) stretching mode for unbound bridging O-H groups remains at 3641 cm^{-1}. This result is in excellent agreement with the PXRD and NPD analyses.

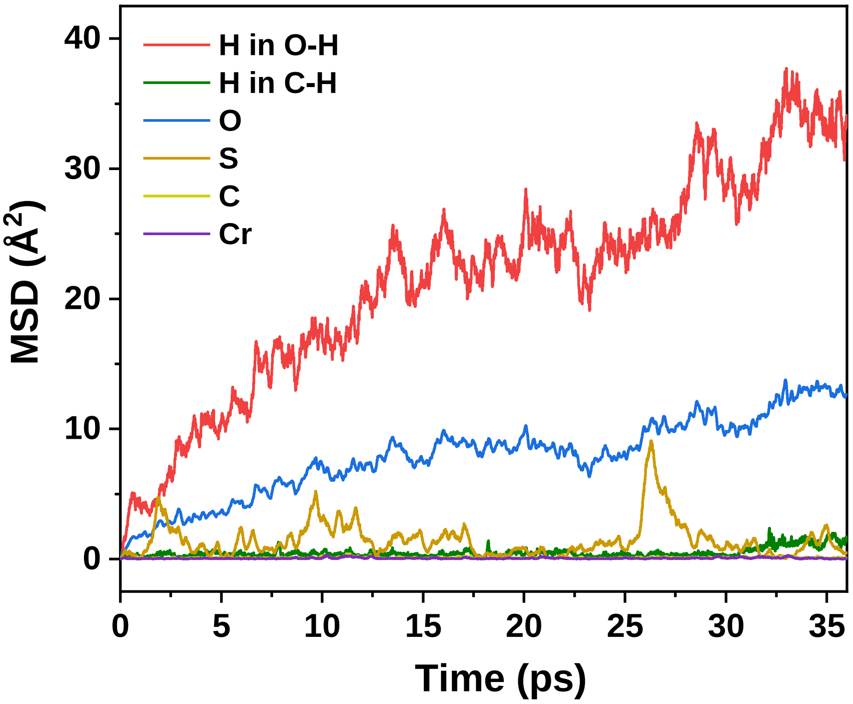

Figure S21. Mean square displacement (MSD) of different elements in MFM-300(Cr)-$SO_4(H_3O)_2$ as a function of time derived from molecular dynamics (MD) simulation. Among all the present elements in MFM-300(Cr)-$SO_4(H_3O)_2$, only protons in the presence of SO_4^{2-} species and H_2O/H_3O^+ are mobile with long range displacement, while other elements either show local motions (such as S/O) or are immobile within the framework (such as Cr or H from linkers). These results are fully consistent with the excellent proton-conduction properties observed in MFM-300(Cr)-$SO_4(H_3O)_2$ and support the experimental results.

4. Supplementary References

(1) Yang, S.; Sun, J.; Ramirez-Cuesta, A. J.; Callear, S. K.; David, W. I. F.; Anderson, D. P.; Newby, R.; Blake, A. J.; Parker, J. E.; Tang, C. C.; Schröder, M. Selectivity and Direct Visualization of Carbon Dioxide and Sulfur Dioxide in a Decorated Porous host. *Nat. Chem.* **2012,** *4*, 887-894.

(2) Briggs, L.; Newby, R.; Han, X.; Morris, C. G.; Savage, M.; Perez-Krap, C.; Easun, T. L.; Frogley, M. D.; Cinque, G.; Murray, C. A.; Tang, C. C.; Sun, J.; Yang, S.; Schroder, M. Binding and Separation of CO_2, SO_2 and C_2H_2 in Homo- and Hetero-metallic Metal-organic Framework Materials. *J. Mater. Chem. A*, **2021**, *9*, 7190-7197.

(3) Coelho, A. A. TOPAS and TOPAS-Academic: an Optimization Program Integrating Computer Algebra and Crystallographic Objects Written in C plus. *J. Appl. Cryst.* **2018,** *51*, 210-218.

(4) Stephens, P. W. Phenomenological Model of Anisotropic Peak Broadening in Powder Diffraction. *J. Appl. Cryst.* **1999,** *32*, 281-289.

(5) Yang, S.; Chen, J.; Chen, X.; Li, W.; Silverwood, I. P. QENS Study of Proton Diffusion in Functional Metal-organic Frameworks. *STFC ISIS Neutron and Muon Source* **2019**, https://doi.org/10.5286/ISIS.E.RB1920005.

(6) (a) Miyatsu, S.; Kofu, M.; Nagoe, A.; Yamada, T.; Sadakiyo, M.; Yamada, T.; Kitagawa, H.; Tyagi, M.; García Sakai, V.; Yamamuro, O. Proton dynamics of Two-dimensional Oxalate-bridged Coordination Polymers. *Phys. Chem. Chem. Phys.* **2014,** *16*, 17295-17304; (b) Mukhopadhyay, S.; Hewer, B.; Howells, S.; Markvardsen, A. A Modern Approach to QENS Data Analysis in Mantid. *Physica B Condens. Matter* **2019,** *563*, 41-49.

(7) Jobic, H.; Theodorou, D. N. Quasi-elastic Neutron Scattering and Molecular Dynamics Simulation as Complementary Techniques for Studying Diffusion in Zeolites. *Microporous Mesoporous Mater.* **2007,** *102*, 21-50.

(8) Kresse, G.; Furthmüller, J. Efficient iterative schemes for ab initio total-energy calculations using a plane-wave basis set. *Phys. Rev. B* **1996**, *54*, 11169.

(9) Bliichl, P. Projector augmented-wave method. *Phys. Rev. B* **1994**, *50*, 17953.

(10) Kresse, G.; Joubert, D. From ultrasoft pseudopotentials to the projector augmented-wave method. *Phys. Rev. B* **1999**, *59*, 1758.

(11) Perdew, J. P.; Burke, K.; Ernzerhof, M. Generalized Gradient Approximation Made Simple. *Phys. Rev. Lett.* **1996**, *77*, 3865.

(12) Klimeš, J.; Bowler, D. R.; Michaelides, A. Chemical accuracy for the van der Waals density functional. *J. Phys., Cond. Matter* **2009**, *22*, 022201.

(13) Yang, F.; Xu, G.; Dou, Y.; Wang, B.; Zhang, H.; Wu, H.; Zhou, W.; Li, J.-R.; Chen, B. A Flexible Metal–organic Framework with a High Density of Sulfonic Acid Sites for Proton Conduction. *Nat. Energy* **2017,** *2*, 877-883.

(14) Pili, S.; Rought, P.; Kolokolov, D. I.; Lin, L.; da Silva, I.; Cheng, Y.; Marsh, C.; Silverwood, I. P.; García Sakai, V.; Li, M.; Tang, C. C.; Yang, S.; Schröder, M. Enhancement of Proton Conductivity in Nonporous Metal–Organic Frameworks: The Role of Framework Proton Density and Humidity. *Chem. Mater.* **2018,** *30*, 7593-7602.

(15) Nagarkar, S. S.; Unni, S. M.; Sharma, A.; Kurungot, S.; Ghosh, S. K. Two-in-One: Inherent Anhydrous and Water-Assisted High Proton Conduction in a 3D Metal–Organic Framework. *Angew. Chem. Int. Ed.* **2014,** *53*, 2638-2642.

(16) Kim, S.; Joarder, B.; Hurd, J. A.; Zhang, J.; Dawson, K. W.; Gelfand, B. S.; Wong, N. E.; Shimizu, G. K. H. Achieving Superprotonic Conduction in Metal–Organic Frameworks through Iterative Design Advances. *J. Am. Chem. Soc.* **2018,** *140*, 1077-1082.

(17) Dybtsev, D. N.; Ponomareva, V. G.; Aliev, S. B.; Chupakhin, A. P.; Gallyamov, M. R.; Moroz, N. K.; Kolesov, B. A.; Kovalenko, K. A.; Shutova, E. S.; Fedin, V. P. High Proton Conductivity and Spectroscopic Investigations of Metal–Organic Framework Materials Impregnated by Strong Acids. *ACS Appl. Mater. Interfaces* **2014,** *6*, 5161-5167.

(18) Kim, S.; Dawson, K. W.; Gelfand, B. S.; Taylor, J. M.; Shimizu, G. K. H. Enhancing Proton Conduction in a Metal–Organic Framework by Isomorphous Ligand Replacement. *J. Am. Chem. Soc.* **2013,** *135*, 963-966.
(19) Wang, S.; Wahiduzzaman, M.; Davis, L.; Tissot, A.; Shepard, W.; Marrot, J.; Martineau-Corcos, C.; Hamdane, D.; Maurin, G.; Devautour-Vinot, S.; Serre, C. A robust Zirconium Amino Acid Metal-organic Framework for Proton Conduction. *Nat. Commun.* **2018,** *9*, 4937.
(20) Otake, K.-i.; Otsubo, K.; Komatsu, T.; Dekura, S.; Taylor, J. M.; Ikeda, R.; Sugimoto, K.; Fujiwara, A.; Chou, C.-P.; Sakti, A. W.; Nishimura, Y.; Nakai, H.; Kitagawa, H. Confined Water-mediated High Proton Conduction in Hydrophobic Channel of a Synthetic Nanotube. *Nat. Commun.* **2020,** *11*, 843.
(21) Slade, R. C. T.; Hardwick, A.; Dickens, P. G. Investigation of H^+ Motion in NAFION Film by Pulsed ^1H NMR and A.C. Conductivity Measurements. *Solid State Ion.* **1983,** *9-10*, 1093-1098.
(22) Taylor, J. M.; Komatsu, T.; Dekura, S.; Otsubo, K.; Takata, M.; Kitagawa, H. The Role of a Three Dimensionally Ordered Defect Sublattice on the Acidity of a Sulfonated Metal–Organic Framework. *J. Am. Chem. Soc.* **2015,** *137*, 11498-11506.
(23) Ponomareva, V. G.; Kovalenko, K. A.; Chupakhin, A. P.; Dybtsev, D. N.; Shutova, E. S.; Fedin, V. P. Imparting High Proton Conductivity to a Metal–Organic Framework Material by Controlled Acid Impregnation. *J. Am. Chem. Soc.* **2012,** *134*, 15640-15643.
(24) Rought, P.; Marsh, C.; Pili, S.; Silverwood, I. P.; Sakai, V. G.; Li, M.; Brown, M. S.; Argent, S. P.; Vitorica-Yrezabal, I.; Whitehead, G.; Warren, M. R.; Yang, S.; Schröder, M. Modulating Proton Diffusion and Conductivity in Metal–organic Frameworks by Incorporation of Accessible Free Carboxylic Acid Groups. *Chem. Sci.* **2019,** *10*, 1492-1499.
(25) Phang, W. J.; Jo, H.; Lee, W. R.; Song, J. H.; Yoo, K.; Kim, B.; Hong, C. S. Superprotonic Conductivity of a UiO-66 Framework Functionalized with Sulfonic Acid Groups by Facile Postsynthetic Oxidation. *Angew. Chem. Int. Ed.* **2015,** *54*, 5142-5146.
(26) Ramaswamy, P.; Wong, N. E.; Gelfand, B. S.; Shimizu, G. K. H. A Water Stable Magnesium MOF That Conducts Protons over 10^{-2} $S\cdot cm^{-1}$. *J. Am. Chem. Soc.* **2015,** *137*, 7640-7643.
(27) Pili, S.; Argent, S. P.; Morris, C. G.; Rought, P.; García-Sakai, V.; Silverwood, I. P.; Easun, T. L.; Li, M.; Warren, M. R.; Murray, C. A.; Tang, C. C.; Yang, S.; Schröder, M. Proton Conduction in a Phosphonate-based Metal–organic Framework Mediated by Intrinsic "Free Diffusion inside a Sphere". *J. Am. Chem. Soc.* **2016,** *138*, 6352-6355.
(28) Zhang, F.-M.; Dong, L.-Z.; Qin, J.-S.; Guan, W.; Liu, J.; Li, S.-L.; Lu, M.; Lan, Y.-Q.; Su, Z.-M.; Zhou, H.-C. Effect of Imidazole Arrangements on Proton-Conductivity in Metal–organic Frameworks. *J. Am. Chem. Soc.* **2017,** *139*, 6183-6189.
(29) Sadakiyo, M.; Yamada, T.; Kitagawa, H. Rational Designs for Highly Proton-Conductive Metal−organic Frameworks. *J. Am. Chem. Soc.* **2009,** *131*, 9906-9907.
(30) Zhai, Q. G.; Mao, C.; Zhao, X.; Lin, Q.; Bu, F.; Chen, X.; Bu, X.; Feng, P. Cooperative Crystallization of Heterometallic Indium–chromium Metal–organic Polyhedra and Their Fast Proton Conductivity. *Angew. Chem. Int. Ed.* **2015,** *127*, 7997-8001.
(31) (a) Karlsson, M.; Matic, A.; Engberg, D.; Björketun, M. E.; Koza, M. M.; Ahmed, I.; Wahnström, G.; Börjesson, L.; Eriksson, S.-G. Quasielastic Neutron Scattering of Hydrated $BaZr_{0.90}A_{0.10}O_{2.95}$ (A=Y and Sc). *Solid State Ion.* **2009,** *180*, 22-28; (b) Borges, D. D.; Devautour-Vinot, S.; Jobic, H.; Ollivier, J.; Nouar, F.; Semino, R.; Devic, T.; Serre, C.; Paesani, F.; Maurin, G. Proton Transport in a Highly Conductive Porous Zirconium-Based Metal–organic Framework: Molecular Insight. *Angew. Chem. Int. Ed.* **2016,** *128*, 3987-3992.
(32) Savage, M.; Cheng, Y.; Easun, T. L.; Eyley, J. E.; Argent, S. P.; Warren, M. R.; Lewis, W.; Murray, C.; Tang, C. C.; Frogley, M. D.; Cinque, G.; Sun, J.; Rudić, S.; Murden, R. T.; Benham, M. J.; Fitch, A. N.; Blake, A. J.; Ramirez-Cuesta, A. J.; Yang, S.; Schröder, M. Selective Adsorption of Sulfur Dioxide in a Robust Metal–Organic Framework Material. *Adv. Mater.* **2016**, *28*, 8705.
